# Floods and rivers: a circular causality perspective

**DOI:** 10.1038/s41598-020-61533-x

**Published:** 2020-03-20

**Authors:** G. Sofia, E. I. Nikolopoulos

**Affiliations:** 1 University of Connecticut, Department of Civil & Environmental Engineering, 261 Glenbrook Rd, Storrs, CT 06269 USA; 2 Florida Institute of Technology, Department of Mechanical and Civil Engineering, Melbourne, FL 32901 USA

**Keywords:** Climate change, Environmental impact, Geomorphology, Climate sciences, Environmental sciences, Environmental social sciences, Hydrology, Natural hazards, Solid Earth sciences

## Abstract

An improved understanding of changes in flood hazard and the underlying driving mechanisms is critical for predicting future changes for better adaptation strategies. While recent increases in flooding across the world have been partly attributed to a range of atmospheric or landscape drivers, one often-forgotten driver of changes in flood properties is the variability of river conveyance capacity. This paper proposes a new framework for connecting flood changes to longitudinal variability in river conveyance, precipitation climatology, flows and sediment connectivity. We present a first step, based on a regional analysis, towards a longer-term research effort that is required to decipher the circular causality between floods and rivers. The results show how this system of interacting units in the atmospheric, hydrologic and geomorphological realm function as a nonlinear filter that fundamentally alters the frequency of flood events. To revise and refine our estimation of future flood risk, this work highlights that multidriver attribution studies are needed, that include boundary conditions such as underlying climate, water and sediment connectivity, and explicit estimations of river conveyance properties.

## Introduction

Flooding poses an ever-present economic, societal^1,2^ and environmental^3^ risk that is likely to increase in the future^4–8^. An improved understanding of historical changes in flood hazard and the underlying driving mechanisms is, therefore, critical for predicting future changes for better adaptation strategies.

Flood estimation and flood management have traditionally been based on flood frequency analysis, following traditional stationary or non-stationary flood distributions accounting for shifts in flood extremes, climate and scale^9–14^. Recent accelerations in population growth, together with changes in the economy and land use patterns have also underlined how humans contribute to the complexity of the flood-river system^15^, as they are inextricably linked to water resources, and are active agents in altering atmosphere, hydrology and geomorphology^16–21^.

Nonetheless, while individual attribution studies linking either atmospheric or landscape drivers to floods are valuable, many times they fail to consider that massive channels widening or narrowing have been recorded^22,23^. These changes in river conveyance are driven by the interactions between hydrology, geomorphology, atmospheric drivers, and human activities, and by the interdependencies of processes at different spatiotemporal scales^24–37^.

**Figure 1:**
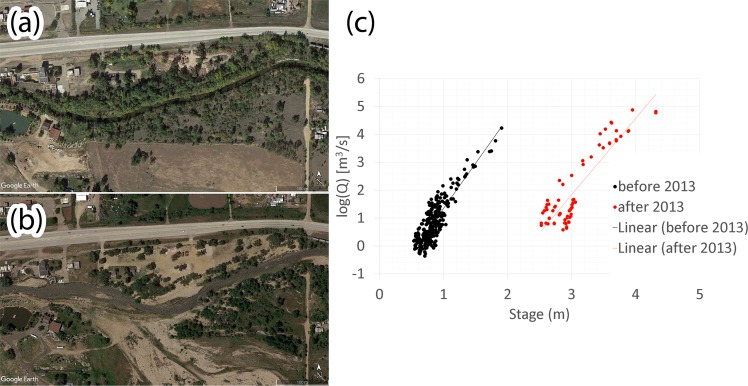
The Colorado’s flood of 2013 changed the geomorphology of the St. Vrain Creek (CO) (**a** in 2012- and **b** in 2017) (Image from Google Earth, 2019 Digital Globe) and altered the stage-discharge relationship (**c**). The map was arranged using ArcGis 10.7 [www.arcgis.com].

Changes in channel geometry at short and intermediate time scales (1 to 100 years) have clear relevance for flood hazard prediction^38^. Fluvial systems function in feedback loops, where processes and their alteration influence one another (Fig. 1), and the cycle may either amplify or reset, continuing over time.

Changes in river conveyance capacity do not impact the amount of water that flows through the river system during a flood, but they have a bearing on the probability of a flood event overtopping the banks and flood defences. However, practice typically assumes that an equilibrium relationship exists between the long‐term average geometry of the channel (i.e., its width, depth, and slope) and the supply of water and sediment to the stream. Although the universality of the equilibrium concept has been challenged (e.g.^39^) it generally approaches the status of canonical law.

Ignoring interdependencies of flood drivers, and the mutual effects of river morphology implicitly promotes a simplified view of the challenges inherent to flood management. Nevertheless, significant effort is still needed to determine how the connections between river conveyance and other flood-drivers look like under different boundary conditions, such as underlying climate, water and sediment characteristics, and in response to internal thresholds and feedbacks.

This paper proposes a framework to investigate the circular causality of channel and floods dynamics, to identify those variables that take part in the behaviour of the river system. It aims to conceptualise and quantify processes and feedbacks between flood properties and rivers. The novelty of the framework is that it considers the broader connectivity between hillslope, floodplain, and stream channel to advance our understanding of flood risk. We propose an analysis that includes historical field-measured flow data, and benefits also from current remotely sensed topographic information.

By characterising the longitudinal river variability together with atmospheric, hydrologic and geomorphologic indices, this research contributes to: (i) a broader discussion about the role of compound drivers in flood trends (ii) identify what catchment‐ and reach‐scale factors influence the interaction among flood drivers, (iii) understand the importance of including river properties and geomorphology in flood analysis.

This study is not meant to develop a general model relating different properties, but rather to document the possible existence of an effect. We offer a starting point upon which to build future research, including a more extensive number of samples and variables. Flood studies typically separate slowly varying boundary conditions of the Earth System (by using static channel properties measured at the outlet of the watershed) from the fast varying hydrological processes (representing the full range of flows). We suggest this approach may no longer be appropriate for estimating future flood risk in a changing world - i.e. for critical infrastructure with a horizon typically within 10–30 yrs.

The remainder of this paper will describe in detail (as independent parameters) the variability of river geometry, connectivity, and flows across basins of interest, as a critical first step in characterising the historical, modern, and future behaviour of these fluvial systems (Chapter 3). Chapter 4 will describe the interdependence of variables. Chapter 5 will show how this transdisciplinary framework making use of remote sensing and historical measured flow allows for a better understanding of flood risk

Ultimately, quantifying the circular causality and understanding the interaction of the hydrologic and geomorphic drivers on flood will allow for revising and refining our estimation of future flood risk and thus leading to better climate adaptation and increase in the resilience of communities and critical infrastructure.

## The Landscape Framework

The investigated landscape framework includes the variables and acronyms summarised in Table 1, and it considers:

(I). Hydraulic Scaling Function(s) (HSF), as power-law functions representing the longitudinal variability of river bankfull geometry. HSFs provide information on the overall river morphology and storage capacity^40^. They offer a quantitative description of how channel width and related properties (depth, velocity) vary with changing discharge along the river course (‘downstream’ hydraulic geometry) and between rivers at a comparable discharge frequency. Specifically, we focus on the coefficients of the power-law that relates bankfull-channel dimensions (width w_bkf_) to drainage area (A_Lidar_) (Chapt 7.1, and 1.2 in supplementary material).
(II). The concept of topographic ‘sediment connectivity’ (IC), understood as ‘the continuity of sediment transfer from a source to a sink’, as a new framework to unravel provenance, pathways and fate of sediments, as well as variability of erosion rates within a catchment^41,42^ (Chapt 7.2, and 1.3 in supplementary material).
(III). River flows and characteristic properties of the daily flow distribution^43,44^, and the decadal trend of exceedance of specific flow quantiles^38^ (Chapt. 7.3 and 1.5 in supplementary material)
(IV). Climatologic characteristics of precipitation, investigated in the form of daily Concentration Index (CI, Chapt. 7.4 and 1.4 in supplementary material). This index offers an indicator for temporal precipitation distribution, and it allows an assessment of seasonal precipitation changes. We will generally refer to ‘climate’ speaking about this specific element.

**Table 1:** Acronyms considered in this study, their meaning and measurement units.

A_Lidar_	km^2^	Drainage area [entire geographical area drained by a river and its tributaries] derived from Lidar^139^
Ph_sec_	—	Physiographic section: geographic areas that share distinct properties like landforms, rock type, and evolutionary history^140,141^
QQ_bkf_	m^3^/s	Field surveyed bankfull discharge at the outlet [the discharge at which a stream first begins to overflow its natural banks onto the active flood plain]
w_bkf_	m	Field surveyed bankfull width [Channel width at Bankfull Discharge] at the outlet
RP_QQbkf_	yr	The return period of the bankfull discharge
D_d_	km/km^2^	Drainage density: length of streams per unit of area
Ω	#	Watershed order: highest stream order of the network, whereas low orders are typical of small tributaries and headwater streams, and higher-order represent rivers transporting more significant volumes of runoff
L_a_	km	Length of the analysed reach: the network segment draining the highest drainage area at each node
L_l_	km	Length of the most extended reach in the network
D_f_	#/km^2^	Drainage Frequency: number of streams per unit of area
HSF		Hydraulic Scaling Function: power-law function that relates bankfull-channel dimensions (width w_bkf_) to drainage area (A_Lidar_). W_bkf_ = *αALidar*^*β*^
α		Scaling coefficient of the HSF
β		Exponent coefficient of the HSF
CI		Climate Concentration Index as provided by^125^. The index reveals the statistic structure of precipitation. High temporal concentration of precipitation is generally linked to the rapid pace of physical processes such as convection in areas with a high degree of insolation and warm seas. The low temporal concentration of rainfall can be interpreted as a consequence of regular patterns (maritime flows or highly recurrent storms).
IC	—	Index of Connectivity evaluated according to^42,142^. It represents the topographic connectivity characteristic of the local landscape, indicating the potential rate of sediment delivery from hillslopes to the investigated channel.
IC_L%_	%	Percentage of the watershed area with low connectivity
IC_H%_	%	Percentage of the watershed area with high connectivity
IC_30_	—	value of IC which is equalled or exceeded for 70% of the flow record (30th percentile) [typical for ‘disconnected’ landscape]
IC_95_	—	value of IC which is equalled or exceeded for 5% of the flow record (95th percentile) [typical for highly connected landscape]
IC_mean_	—	Mean IC value
IC_cv_	—	Coefficient of variation of the IC: the ratio of the standard deviation to the mean
Q_mean_	m^3^/s/km^2^	Mean specific daily discharge (daily mean normalised by catchment area)
Q_bkf_	m^3^/s/km^2^	Specific Bankfull discharge (bankfull discharge normalised by catchment area)
Q_cv_	m^3^/s/km^2^	Coefficient of variation of the daily flows: the ratio of the standard deviation to the mean
Q_30_	m^3^/s/km^2^	daily flow which is equalled or exceeded for 70% of the flow record (30th percentile) (normalised by catchment area): represents ‘base flow’ levels
Q_95_	m^3^/s/km^2^	daily flow which is equalled or exceeded for 5% of the flow record (95th percentile) (normalised by catchment area): represents extreme flows
xQ_95_	%/decade	Decadal trend in exceedance of Q95
xQ_30_	%/decade	Decadal trend in exceedance of Q30

### River geometry

**Figure 2:**
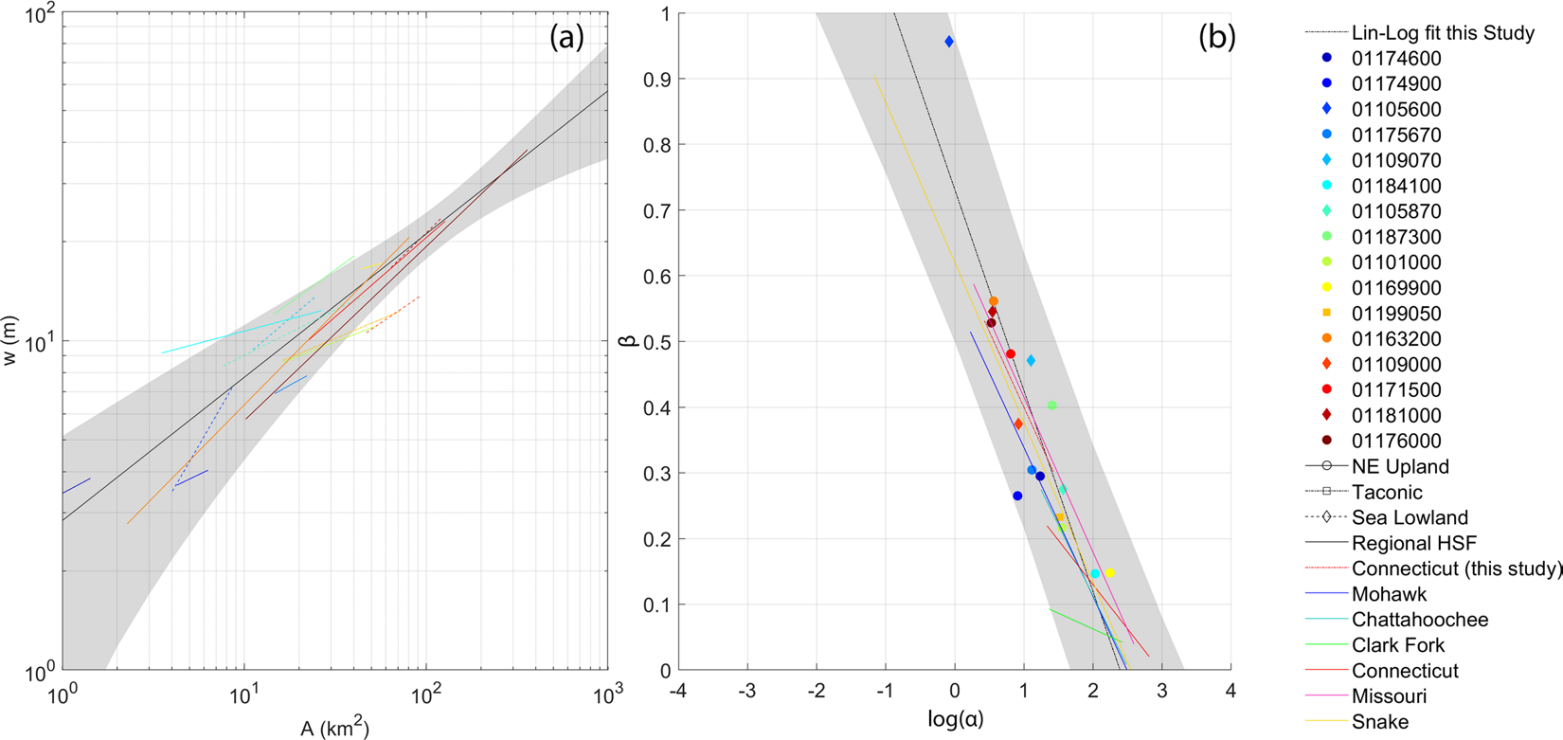
The wide range of flows that each watershed experiences leads to the formation of a unique degree of adjustment of bankfull channel geometry (α and β). The variability of the coefficients across watersheds suggests that there are a set of width-drainage area values (and therefore, possibly, discharge) that are shared by cross-sections across watersheds (**a**), and that paired scaling and exponents exhibit very strong semi-log relationships (a straight line when one variable is logged and the other variable is not) over the investigated region. Displayed plots show: (**a**) local HSF for each watershed as compared to the regional regression from field survey (Dataset S2 supplements): (**b**) coefficients of the HSF (α and β) in the log-linear domain, with the derived regression as compared to those of the Snake, Missouri, Connecticut, Clark Fork, Chattahoochee and Mohawk rivers ^60^. Points and HSF provided by our study are colour-coded according to the watershed drainage area (from blue to red for increasing values).

Current studies in geomorphic drivers of floods^38^ focus on variability considering channel measurements at a single site. Yet, the channel width is related in a remarkably regular way to bankfull discharge as it varies along the river course^45–47^. The average coefficients obtained for the rivers from Lidar (Fig. 2a) are α = 3.6 ± 2.3 and β = 0.39 ± 0.21 and they are in line with the regional HSF reported in the literature [1.0 < α < 5.83 and 0.1 < β < 0.6]^48–54^. They also well capture field-surveyed bankfull widths at the outlet [standard deviation of percentage error ±10%, average absolute error ~0.60 m, standard deviation of absolute errors ± ~2 m], with higher accuracy (15%) than downstream regressions generally obtained from field survey (around 30%).

By aggregating local HSF parameter pairs from many distributed locations and plotting the coefficients in a log-linear domain^55^, it is possible to identify a regional trend that does not follow a downstream direction [slope = −0.70 and intercept = 0.73 (RMSE = 0.013 m, R^2^ = 0.78) (Fig. 2b)]. Despite being derived using drainage area rather than discharge, the log-linear equation for this study well overlaps with literature values^55^, presenting, however, steeper slopes for the watersheds within the Connecticut river domain (Fig. 2b). These differences pose more emphasis on exploring the correspondence between HSF and discharge-area relationship^56^ to investigate the causes for a significant departure from linearity in the log-domain.

Flow variation plays a vital role in channel evolution and maintenance. The wide range of flows that each watershed experience leads to the formation of unique bankfull channel geometry. In turn, observing the degree of the adjustment process (coefficients of the HSF, Fig. 2a) implicitly contains information about the combined effects of erosion and deposition of both the channel and the floodplain, which should be considered as parts of a single unit, and further contains a description of the variability of flood-river interactions along the river course. Comparing HSF coefficients for the considered watersheds (Fig. 2) suggests that there are a set of width-drainage area values that are shared by cross-sections across watersheds. The range of values highlights the existence of a ‘geomorphological allometry’^57^ [static changes in stream channel size, relative to similar values of drainage area, at a variety of watershed scales]. Generalising, the coefficients of the HSF represents the implicit signature of additional variables, other than drainage area, including flow regimes, regional climatic and physiographic factors, geological characteristics, the responsiveness of the catchment, and human activities across the investigated watersheds.

With field surveys, the uncertainty in the determination of a bankfull width and discharge is significant^58^. As well, the determination of bankfull discharge frequency is not easy because the classic hydrological methods developed for floods reach their limits. For flood hazard estimation, the proposed Lidar-based analysis leads to the conclusion that only one of the two traditional calibration parameters of the HSF may suffice to infer bankfull widths from the drainage area if a regional trend is well constrained. Channel geometry alters flood hazard locally^38^, yet the proposed investigating HSFs across watersheds suggests that transient changes in channel dimensions at a point are minor compared to the variability of downstream trends. Investigating the relationship between HSF coefficients and other flood drivers will allow i) investigating the response of a river system to climate and landform settings (sediment connectivity) and ii) diagnosing the causal agency of river channel and flow properties.

### Sediment connectivity

This chapter evaluates the concept of sediment connectivity as a proxy for the processes involved in sediment transfer across multiple scales.

**Figure 3:**
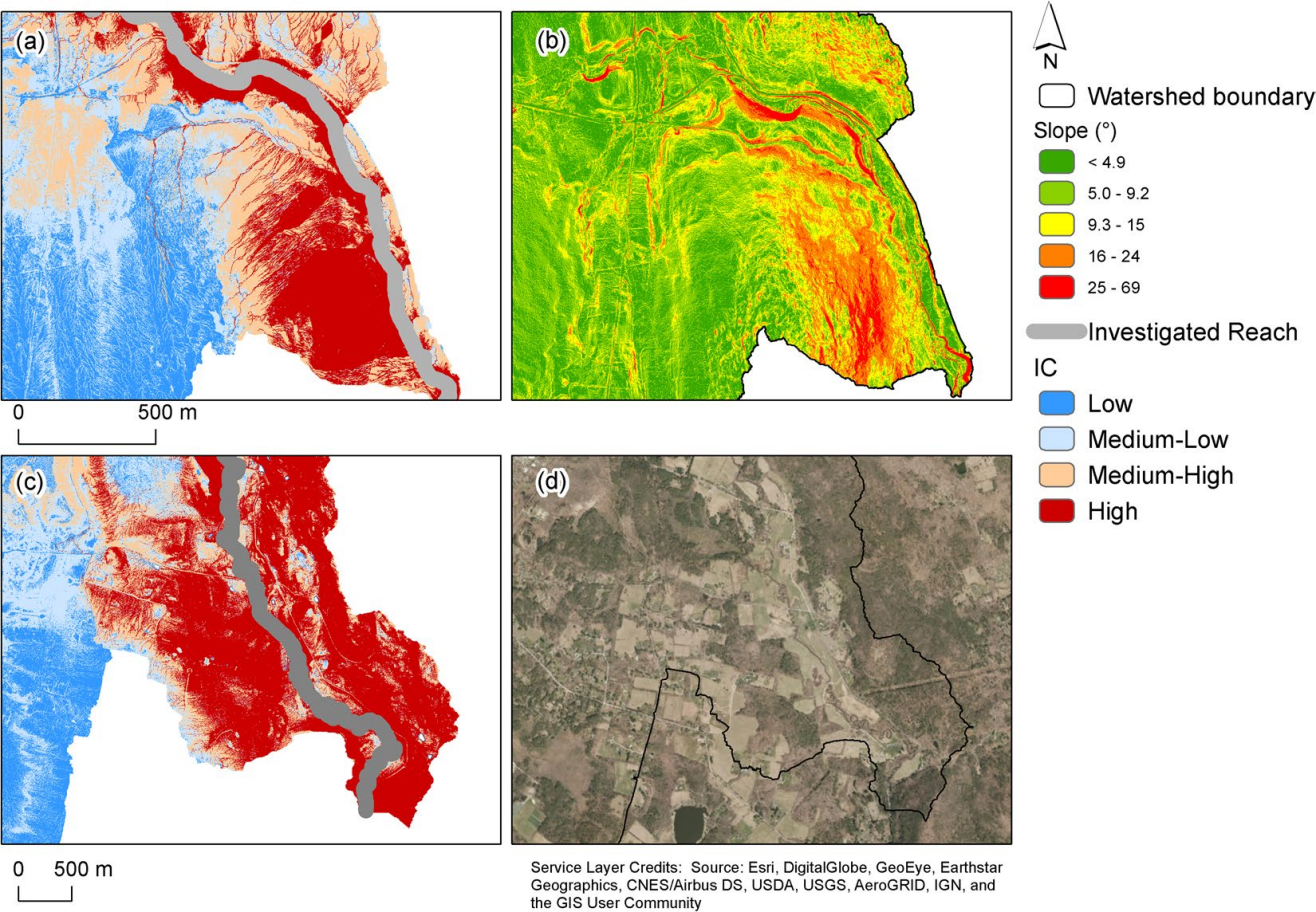
Various landscapes features [high slope (**b**), and agricultural fields (**d**)] increase landscape connectivity (more extensive areas with high values of the connectivity index -IC-), determining the amount of rainfall to be transformed into runoff and sediment to be possibly delivered to the river reach, and therefore influencing flood risk. Examples are shown for the Cadwell (**a,b**) and Salmon Creek (**c,d**) watersheds. The figure was arranged using ArcGis 10.7 [www.arcgis.com]. Slope was evaluated using customized scripts in Matlab 2018b [https://www.mathworks.com/release2018b], while the connectivity index was derived using the stand-alone tool by^133^.

The watersheds present different degrees of connectivity to the investigated reach (Fig. S2 supplements, Fig. 3), from fully linked to fully unlinked [low-connected (low and medium-low) areas covering from 25% to 70% and highly connected areas (Medium-High and high) from 29% to about 70%]. More efficient sediment connectivity is observed in smooth steep catchments rather than from dissected or stepped landscapes [as also in (Baartman *et al*., 2013)]. Nevertheless, various landscapes features, such as drastic changes in slope (Fig. 3b, 01174900 –Cadwell Creek near Belchertown, MA) or human activities (01199050 – Salmon Creek, CT, Fig. 3d) increase landscape connectivity (connectivity index -IC- within the high cluster, as shown in Fig. 3a,c respectively), determining the amount of sediment to be potentially delivered to the river reach, and therefore, possibly, influencing flood risk.

**Figure 4:**
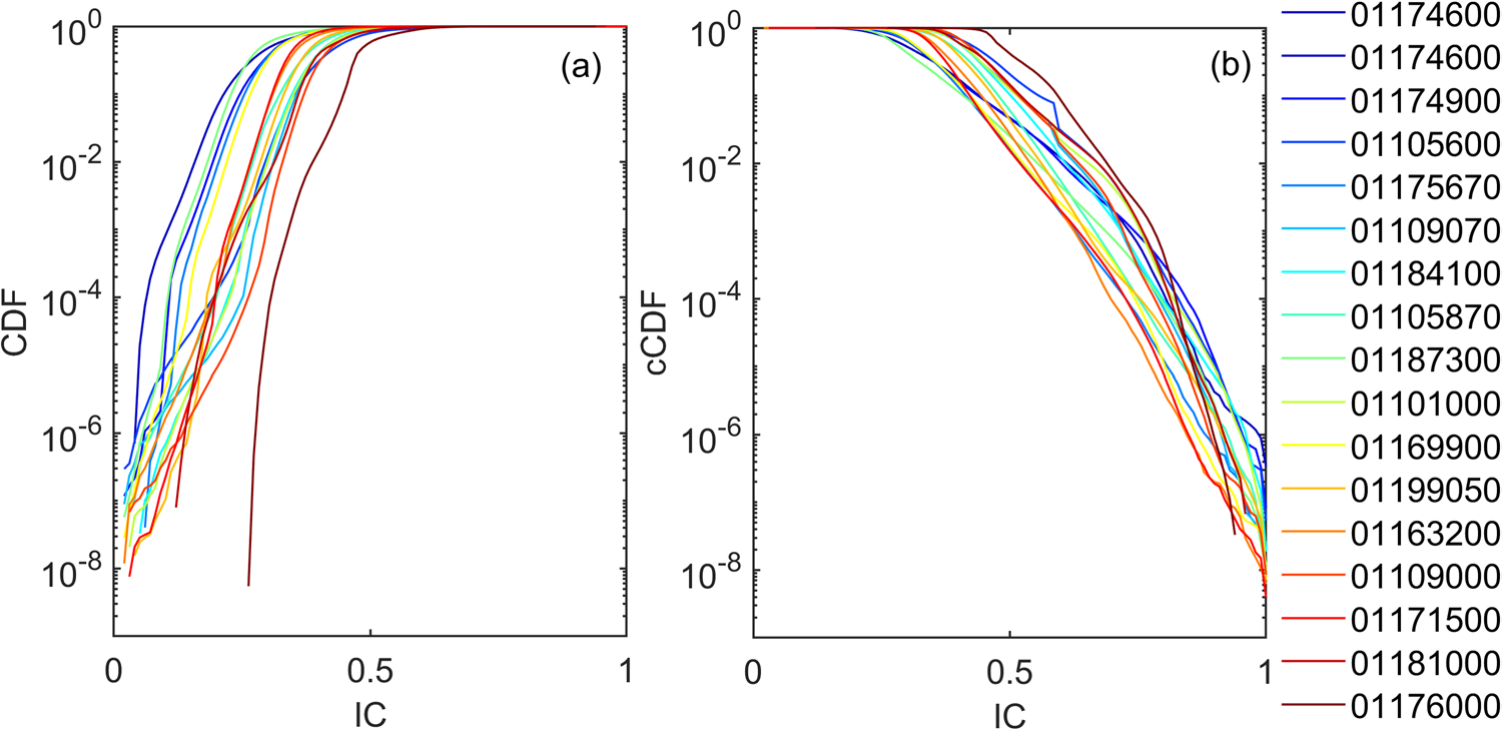
Cumulative (CDF, (**a**) and complementary cumulative distribution (cCDF, **b**) function for the connectivity index (IC), obtained for each watershed (colour-coded according to the watersheds drainage area, from blue to red for increasing values). Within each watershed large portions of the landscape tend to present low values of connectivity (**a**), while generally smaller extents show comparable high connectivity (**b**). Clusters of disconnected (**a**) or connected (**b**) landscapes can be seen [decreasing slope in the CDF or cCDF, where the gradient increases again later], highlighting the existence of diverse (across watersheds) but consistent (within watersheds) morphodynamics units, acting as sinks or source of sediments.

Notwithstanding the intra-watershed variability, within each watershed, large portions of the landscape tend to present low values of connectivity (they are exceeded >70% of times, Fig. 4a). Clusters of disconnected landscapes can be seen [decreasing slope in the Cumulative Distribution Function -CDF-, where the gradient increases again later], highlighting the existence of diverse (across watersheds) but consistent (within watersheds) morphodynamics units, acting as sinks of sediments. The high variability of connectivity appears independent from the scaling (drainage area).

The complementary CDF (cCDF, Fig. 4b, that describes the probability that a particular value for a random variable will be exceeded) highlights that fully connected landscapes generally cover the lowest extent of the watershed. Nevertheless, clusters of high connectivity values, possibly representing various morphodynamics units, can be seen for the connected landscape as well (Fig. 4b, decreasing slope in the cCDF where the gradient increases again later). Whether these clusters are proximally located to potential sediment sources upstream, channel aggradation might be likely if sediment supply and runoff increases from upstream areas. Channel bed aggradation might also induce further channel avulsions as it forces floodwaters across the floodplain.

Notwithstanding essential assumptions of the effectiveness of local measures on inferring catchment related erosion rates, monitoring potential sediment delivery at a river section can provide an order of magnitude of erosion and depositional processes within a catchment^59,60^. The presence and spatial configuration of different linkages and blockages as well as their capacity to store or remove sediment within different spatial scales, as highlighted by Figs. 3 and 4, determines the efficiency of the sediment cascades^61,62^ and the potential effects on flood hazard. We can expect catchments with a higher rate of connectivity, and in sediment-prone landscapes, to be more prone to dramatic floodings. When and if sediments are delivered, a single flooding happening at the local scale can be affected by the presence of sediments, which are moved by water from the main channel and deposited along the floodplains, changing the channel capacity and therefore driving to a possible increase in the flood risk and the residence time of water on floodplains^63^.

### Flows

**Figure 5:**
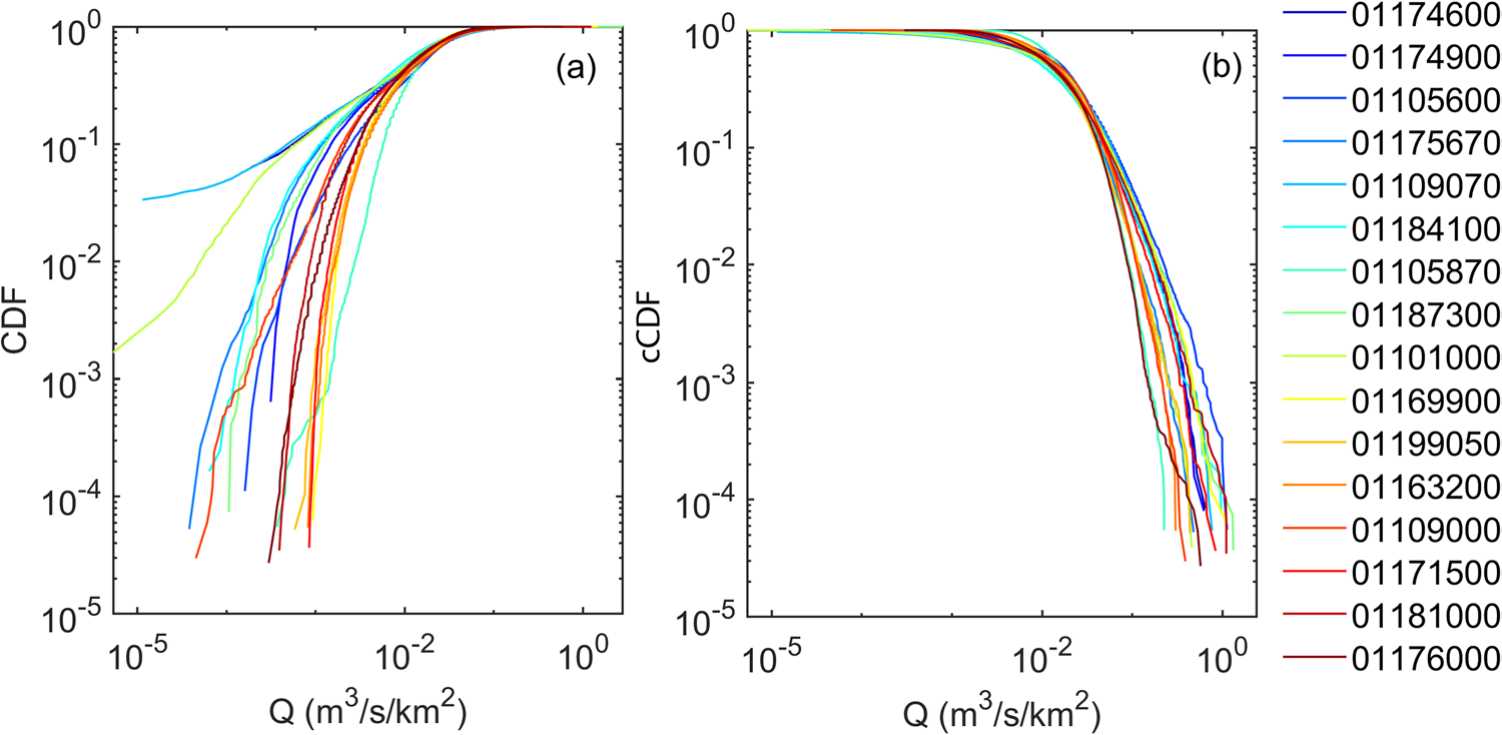
Cumulative (CDF, (**a**) and complementary cumulative distribution (cCDF, (**b**) function for the specific discharge, obtained for each watershed (colour-coded according to the watersheds drainage area, from blue to red for increasing values). The CDF highlights the existence of ‘wetter’ catchments (i.e. 01187300 and 01109070) with greater ability to maintain soil moisture, store precipitation and thus increase the base flow. The cCDF provides a compact signature of a catchment functioning that appears to be not related to changes in watershed scale (warm and cold colours in (**b**) are not well separated), but rather to elements such as land-use (i.e. urban areas and agricultural lands rather than forested watersheds).

The distribution of daily specific discharge (Fig. 5) for the analysed catchments presents considerable variability in low and high flow values. Low flow conditions exhibit a distinct separation between the cold and warm colours (Fig. 5a), suggesting that catchments with larger drainage area exhibit higher values of specific discharge. Watersheds showing a CDF highly different from the others (i.e. 01187300 and 01109070 in Fig. 5b) present lakes and wetlands, and coarse soils. These ‘wetter’ catchments might have a damping effect on water variability and dry periods, and they have a greater ability to maintain soil moisture, store precipitation and thus increase base flows^43^.

The cCDF (Fig. 5b), as flow duration curve, provides a compact signature of a catchment functioning. The analysis shows that flow signature is not related to changes in watershed scale (warm and cold colours in Fig. 5b are not well separated), but rather to elements such as land-use (i.e. urban areas and agricultural lands rather than forested watersheds).

Collectively, the information derived from the comparison of the specific discharge distributions among watersheds testifies that runoff generation (e.g. surface and subsurface contributions) varies considerably across the different catchments.

**Figure 6:**
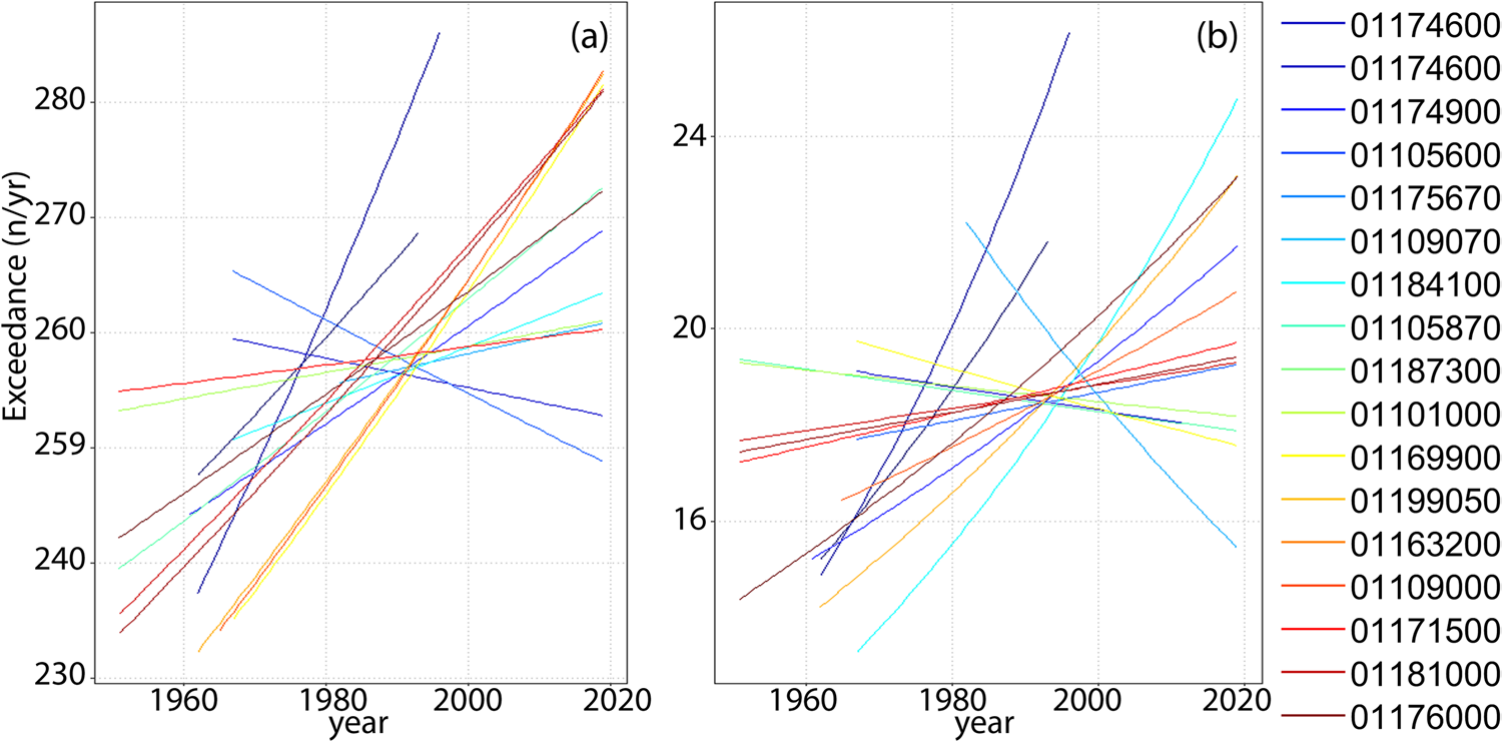
Flow frequency mean-unbiased exponential trend (in units of exceedance of Q30 (**a**) and Q95 (**b**), n/yr) versus year obtained for each watershed (colour-coded according to the watersheds drainage area, from blue to red for increasing values). The slope of each curve identifies increasing (positive) or decreasing (negative) trends during the years. These curves demonstrate that nonstationarity in flood frequency is common. Trends in exceedance of low (**a**) and extreme flows (**b**) are widespread across scales and landscape units, challenging existing paradigms of flood frequency analysis and channel design.

Almost 90% of the sites (14/16) show increasing trends in low-flow exceedance (Fig. 6a). Nearly 70% of them (11/16) show significant increasing trends in exceedance of extreme flows (Q95) as well. Flow frequency trends for extreme flows (an average decadal increase of 6.6% and decrease of 3.0%, Fig. 6b) are typically larger than that of low-flows (an average decadal increase of 2.2% and decrease of 0.9%, Fig. 6a).

About 30% of the watersheds shows asynchronous trends in flow exceedance (decadal increasing in exceedance of low-flows vs decadal decrease in extreme flow exceedance). Statistically significant low-flow trends are nearly three times more common than statistically significant high flows (43% vs 12%), suggesting that trends in low flows might be more widespread and easier to detect over decadal time scales.

These findings suggest that flood hazard is generally nonstationary, and undermines most efforts to characterise flood hazard over decadal time scales by fitting theoretical probability distribution functions to historical flood records^38^.

## The Interdependence of Variables

Connecting changes in river flood hazard to its drivers requires two main assumptions: (i) the resulting signal is a mixture of component signals, and (ii) the patterns of the component signals are known to some degree^14^.

For this chapter, the observed signal will be the trend in exceedance of Q_95_ (xQ_95_), while the component processes are flow properties (Q), landscape properties (connectivity -IC-, physiographic section Ph_sec_), climate (CI) and river geometry (α,β), whose correlation can be considered to be a fingerprint associated with many catchments within the region. As a consequence, these variables can be used for regional flood change investigation, rather than for attribution in individual catchments.

**Figure 7:**
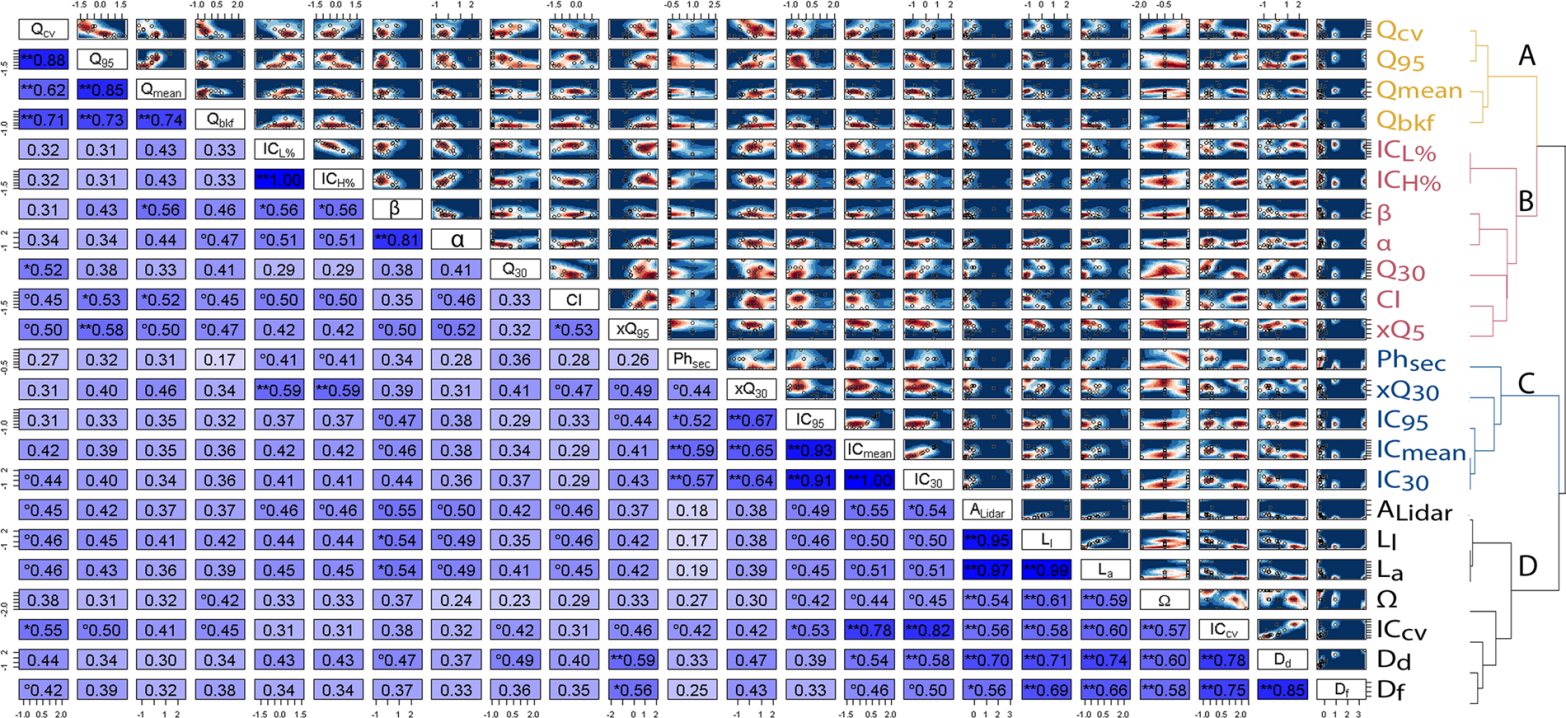
Atmospheric (CI), landscape (IC, Ph_sec_), river (α,β) and flow (Q, xQ) properties are correlated to different degrees (values of correlation -dCor- close to 0 represent no correlation, and 1 represent perfect correlation) [Lower triangular matrix in the image; shades of blue highlights the correlation strength, from weak (light) to strong (dark)], with some strong non-linear dependence emerging between specific variables [dCor values marked with ** are statistically significant at the 0.05 level, values marked with * are significant at the 0.1 level, values marked with ° are significant at the 0.2 level.] The interaction between variables [open circles in the upper triangular matrix] reinforces or offsets the decadal trends of exceedance of extreme flows [upper triangular matrix; shades of red represent progressively increasing decadal trend, white represent no trend, and shades of blue represent decreasing trends]. We used a kernel density estimation (KDE^138^,) to display in a non-parametric way the decadal trend for each 2 way combination of landscape indicators, using the start positions of each variable within the scatterplot. The correlation strength allows to define cluster of variables (dendrogram) that are more closely related. Elements that are merged at a lower height (shorter branches in Fig. 7) are more similar (dependent) than Elements that merge at greater height. It is possible to identify four separate clusters, including flow properties (**A**), reach properties, climate and flood hazard (**B**), morphodynamics properties (**C**), and scale-dependent parameters (**D**). Figure was arranged using customized scripts on Rstudio Version 1.2.1335. See *Data and software availability* for details.

Flow, landscape, climate and river properties identify dominant processes in flood hazard, and the strength of correlation amongst them (lower triangular matrix in Fig. 7, where values of correlation -dCor- close to 0 represents no correlation, and 1 represents perfect correlation) determine the strength, speed and spatiotemporal variability of the rainfall-runoff response. As a consequence, the interaction between them reinforces or offsets the decadal trends of exceedance of extreme flows (xQ_95_, upper triangular matrix in Fig. 7, where shades of red represent progressively increasing decadal trend, white represent no trend, and shades of blue represent decreasing trends).

One characteristic fingerprint emerging is that of ‘drainage allometry’: drainage density, drainage frequency, watershed order, the length of the analysed channel, and length of the longest trunk of the network presents strong statistically significant changes relative to similar values of drainage area, at a variety of watershed scales (Fig. 7).

Flow properties also well represent a specific fingerprint, displaying strong correlations (Q_95,_ Q_mean,_ Q_CV,_ Q_bkf_) (Fig. 7).

Further fingerprint is given by sediment connectivity at the catchment scale (IC_mean,_ IC_30,_ IC_95_) and physiographic settings (Ph_sec_). The dispersion of sediment connectivity (IC_cv_), however, appears to be mostly related to scale (A_lidar_), with a nonlinear behavior and smaller CVs in larger catchments (Fig. 7).

Reach scale connectivity (the percentage of high or low connection to the investigated stream IC_H%_ and IC_L%_) is strongly related to the trend in low flows (xQ_30_) and channel geometry (a,b).

The distance correlation confirms the non-linear nature of the relationship between the coefficients of the HSF (α and β, as shown previously in Fig. 2). The HSF coefficients also appear to be related with some network properties, connectivity and the decadal trend in extreme flows (xQ_95_).

Low-flows exceedance trends are highly correlated with landscape properties (connectivity -IC-, physiographic section Ph_sec_). Exceedance of extreme flows (xQ_95_) is conversely related to some network parameters (drainage density and frequency), climate (CI) and HSF.

It is crucial to notice, however, that it should never be concluded there is ‘no association’ just because a p value is larger than a threshold^64^. The use of the correlation distance in a clustering approach offers a different angle to observe the relationships among parameters. Fingerprints that are merged at a lower height (shorter branches in Fig. 7) are more similar (dependent) than fingerprints that merge at greater height. One can observe, for example, that the flow properties (A in Fig. 7) cluster together, and that reach properties, climate and flood hazard (B in Fig. 7) also cluster together, but these two clusters are well separated and not merged until the second to last step when there are four clusters.

This highlights that, if on the one hand, flood hazard signals essentially reflect observed changes in precipitation^13,65–68^, reach-scale connectivity and channel geometry might also adjust to rainfall erosivity, as well as to the repercussions of climate on water cycle.

One can also observe that morphodynamics properties (C in Fig. 7, connectivity -IC-, physiographic section -Ph_sec_- and trends in low-flows) cluster together, as do the drainage allometry signature together with catchment scale connectivity dispersion (D in Fig. 7).

The morphodynamics properties cluster confirms how catchments geologic characteristics impact infiltration and thresholds of overland flows, modifying the landscape and determining connectivity to the network. As well, it highlights how feedbacks between landscape components (connectivity, physiographic regions) are a critical component of watershed discharge^69^.

The drainage allometry signature cluster confirms the fact that river networks have long been recognized as possessing self-similar structures over a considerable range of scales^70–75^.

**Figure 8:**
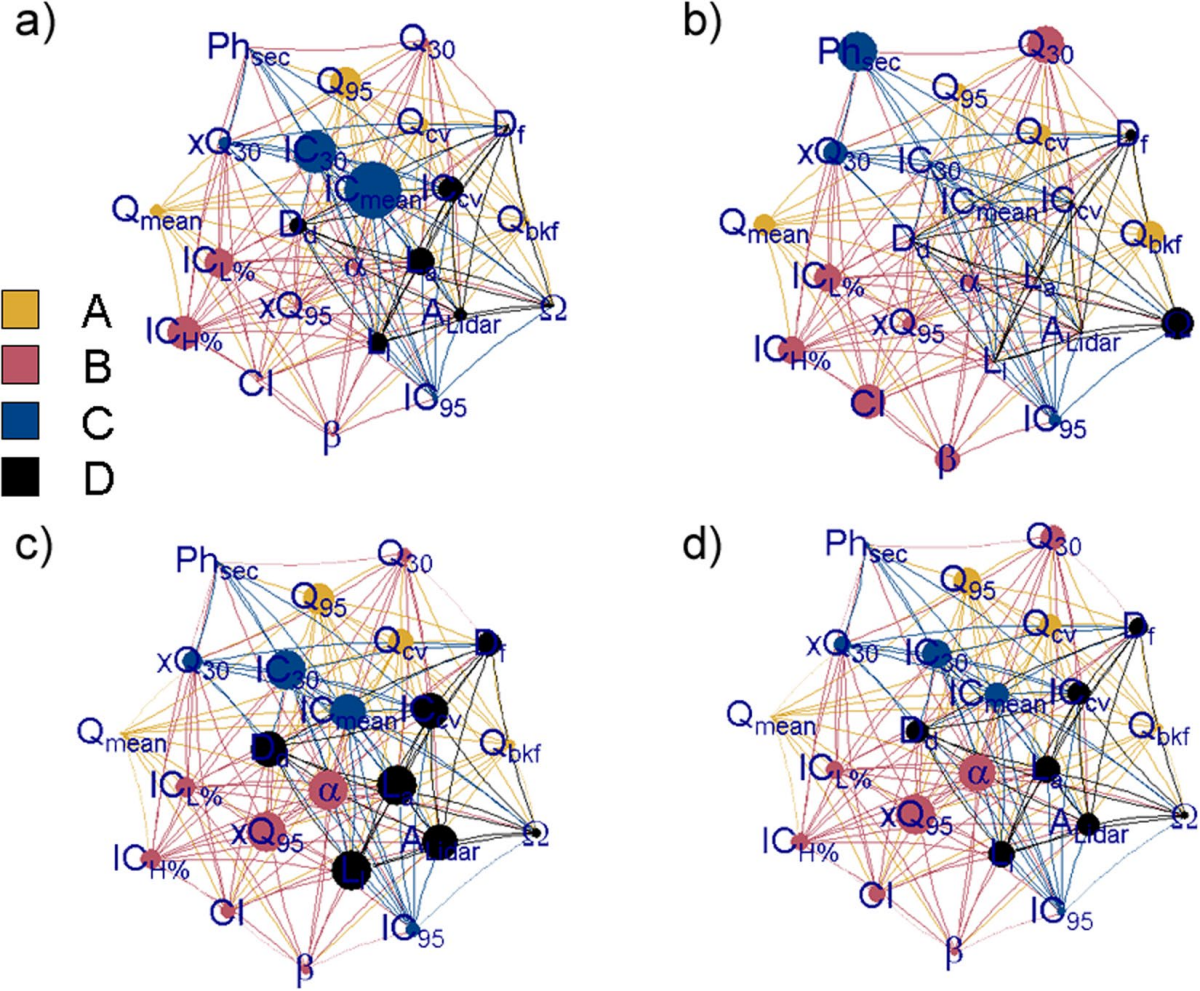
Components of the various clusters (Flow properties (A), reach properties, climate and flood hazard (B), morphodynamics properties (C) and scale-dependent parameters (D)) assume different roles within the network depending on the centrality measure considered [larger importance is highlighted by symbols with sizes proportional to the centrality measure: betweenness (**a**), closeness (**b**), degree (**c**), and strength (**d**)]. Mean connectivity and rate of dis-connectivity (ICmean, IC30 in a) have more control over the network, because more information pass through these nodes (highest betweenness). Physiographic properties, low-flows and climate (Ph_sec_, Q_30_, CI in b) have more direct influence on other vertices (lowest closeness). The rate of channel adjustment, decadal trend in extreme flows and the rate of disconnectivity (slope of the HSF β, xQ_95_ and IC_30_ in (**c**)) are connected to lots of nodes at the heart of the network (highest degree), however the decadal trend in extreme flows (xQ_95_, **d**) is the node with the higher level of correlation (highest strength). Figure was arranged using customized scripts on Rstudio Version 1.2.1335. See *Data and software availability* for details.

The identification of fingerprint clusters (Fig. 7) offers the basis to identify multiple mechanisms that typically contribute to flood risk. These mechanisms can be characterized as a set of interconnected components (or network, Fig. 8, Table 2). Each objects (e.g., morphodynamics properties and scale-dependent parameters in Fig. 7), regimes (e.g. flow properties in Fig. 7) or phenomena (e.g. reach properties, climate and flood hazard in Fig. 7) are connected by fluxes of matter and energy, feedbacks, spatial or temporal sequencing or adjacency, statistical correlations, and process-response relationships.

**Table 2:** Flow properties (A), reach properties, climate and flood hazard (B), morphodynamics properties (C) and scale-dependent parameters (D) presents present different roles in the network (most important roles highlighted in bold). Clusters with higher betweenness centrality (C) have more control over the network, because more information pass through their nodes. Their role is related to the network’s connectivity, in so much as high betweenness vertices have the potential to disconnect graphs if removed. Assuming that vertices can only pass messages to or influence their existing connections, a clusters with low closeness centrality (B) means that are directly connected or “just a hop away” from most others in the network. In contrast, clusters in very peripheral locations may have high closeness centrality scores (D), indicating the high number of hops or connections they need to take to connect to distant others in the network. Degree centrality shows how many connections a cluster has. They may be connected to lots of nodes at the heart of the network (D), but they might also be far off on the edge of the network. Clusters with high strength (B) present connections with a higher level of correlation.

	A	B	C	D
	*flow properties*	*reach properties, climate and flood hazard*	*morphodynamics properties*	*scale-dependent parameters*
betweenness	0.250	0.176	**0.411**	0.257
closeness	0.532	**0.358**	0.615	0.821
degree	0.438	0.571	0.550	**0.786**
strength	0.348	**0.589**	0.415	0.525

A first point that the network allows to discuss is the importance of network components for the whole network (betweenness). Clusters with higher betweenness centrality (such as the morphodynamics properties, C, in Figs. 7, 8a and Table 2) have more control over the network, because more information passes through their nodes. Among the nodes within this cluster, mean connectivity and rate of dis-connectivity (IC_mean_, IC_30_ in Fig. 8a) emerge as prominent. Their role is related to the network’s connectivity, in so much as high betweenness vertices have the potential to disconnect graphs if removed. Describing sediment connectivity in a landscape allows to consider the spatial organization of various physiographic units and their contribution to sediment production, transport and deposition, and emphasizes the importance of landscape features to enhance the transfer of water and sediment towards flooded areas.

Further aspect to consider relates to paths through the network, i.e., their length, cost, or routing capacity (closeness in Fig. 8b, Table 2). The shortest (geodesic) path is that which contains the smallest number of edges; it can also be a least-cost path in terms of the lowest cumulative cost or friction. Reach properties, climate and flood hazard are directly connected to most others in the network (low closeness, Fig. 8b, Table 2). Amongst the nodes of this cluster, physiographic properties, low-flows and climate (Ph_sec_, Q_30_, CI in Fig. 8b) have more direct influence on other vertices (lowest closeness).

In this complex network, it is important to identify what nodes are connected or reachable from one another. The degree of a node is the number of edges that connect it to other nodes. If the edges are weighted, and the weight attribute is considered in adding up incoming and outgoing edges, the corresponding node property is its strength. Scale-dependent parameters (D in Figs. 7, 8c, Table 2) may be (in average) connected to lots of nodes at the heart of the network, but they might also be far off on the edge of the network. However, the rate of channel adjustment (slope of the HSF β), decadal trend in extreme flows (xQ_95_) and the rate of disconnectivity (IC_30_ in Fig. 8c) are the nodes with the highest degree. The same nodes have increased importance in terms of strength (Fig. 8d), with the decadal trend in extreme flows (xQ_95_, d) being the node with the higher level of correlation (highest strength).

Centrality depends on the way the graph-theoretical model of flood drivers is constructed, although even the simplest network representation, not taking directionality of flows into account, still provides a coarse-grained assessment of the most important nodes according to their contribution to the regional variability, and highlights how trends in flood hazard (xQ_95_) are the element connected to lots of nodes at the heart of the network. Their role implies that spreading power is determined by both the degree of the node and the degree of its neighbors (nodes of the reach properties, climate and flood hazard cluster). This implies that changes in climate, channel geometry and the degree of connectivity to the investigated river are prominent, and they have multiple alternative ways to modulate and transfer changes to flood hazard.

The observed network (Fig. 8) is (intentionally) undirected. Future analysis of network components should include directional edges, to identify sets of connected entities within a database containing many watersheds distributed across areas and at different points in time^76^. Similar networks/sub-networks, as appropriate, should also be formulated for different temporal and spatial scales of interest. This would provide a framework for assessing the historical contingency and stability of the network, and would allow to understand how contemporary features are sensitive to changes in an environmental characteristic of the past.

## River and flood: processes and feedbacks

The investigated landscape shows geomorphological allometry (Fig. 2), clusters of morphodynamics units (Fig. 4), signatures of a catchment functioning (Fig. 5) that are linked to trends in exceedance of low and extreme flows (Fig. 6) widespread across scales and landscape units, challenging existing paradigms of flood frequency analysis and channel design.

Hydraulic forces, climate or geomorphic variables, if considered alone, are not sufficient to explain the flood response (Figs. 7 and 8) [see e.g.^4,29,77–80^].

Non-linear relationships, depending on which processes dominated under a particular hydrologic regime, emerge across watersheds (Fig. 7)^4,14,81^. The long line of recent major floods across the world highlights risks of new climate reality^5,6,13,82^. Nevertheless, climate impact on flood hazard is complex and depends on the river flood generation mechanism, and difficulties exist in disentangling the climatic component from substantial natural variability and direct human impacts on flows^78,83^.

Looking at climate (Fig. 7), higher precipitation concentration (CI) represented by greater percentages of the yearly total precipitation in a few rainy days, has the potential to cause flood and it is indeed correlated with higher discharge (Q_95_) and positively correlated with increasing trends in flood hazard (xQ_95_). Nevertheless, increasing trends in flood hazard also depend on the overall river morphology and storage capacity, as well as landscape connectivity (cluster C in Figs. 7, 8, Table 2). Observing climate together with other variables shows high temporal concentration of precipitation (high CI) is related to increasing trends in flood hazard especially when channels narrows (decreasing exponent of the HSF), or if sediment connectivity increases CV.

More information pass through the nodes representing sediment connectivity (highest betweenness). Sediment supply can change with altered connectivity upstream and changes in hillslope–channel coupling^60,84,85^. Seasonal timing and sequence of events can affect the watershed response: extreme rainfall events can lead to significant soil loss^86,87^, and modify depression storage changing the connectivity of overland flow^88^, with implications for downstream flood risk and sediment‐related flood damages^33,63,89^.

Considerable narrowing and decrease in channel conveyance over short timescales might substantially increase the potential for floodplain inundation, as increasing trends in exceedance of extreme flows are registered for rivers with lower values of the HSF coefficients (Fig. 7). This might be even more evident for landscapes where rivers present lower capacity (small exponent of HSF) and high sediment delivery potential to the investigated channel (high levels of IC_H%_) (Fig. 7), or when watersheds present a very extensive network (high D_d_ in Fig. 7) but with low level of conveyance in their main reach (low b values).

Among the catchment-scale properties, drainage network structure also emerged as having a critical role (drainage density, drainage frequency, Figs. 7, 8). One must consider that river planform structure is one of the elements mostly modified in anthropogenic landscapes^28–31^. The centrality of these parameters in the graph, and their correlation with decadal trends in flood hazard, hints to the fact that these changes that might happen relatively quickly compared to the long-standing life of a river, might be a very sensitive trigger to further flood-feedbacks.

From a network perspective (Fig. 8), longitudinal channel adjustment might be able to disperse changes quickly to many other components of the network (low closeness and high strength/degree). This strengthen the idea that river geometry is not a static collector that accommodate and convey (or otherwise) the runoff generated by precipitation distributions. Instead, it dynamically adjusts to/and adjusts flows, which means that altered channel properties due to changes in drivers will, in turn, alter the risk of future flooding (Fig. 1). One should consider that channel geometry adjusts to climate^34,90,91^ or anthropogenic pressure^15,29,92^, and according to channel-maintaining^93^ and channel-changing discharges^26,62,94,95^. This form of feedback means that channel geomorphic response could cause a legacy of altered flood risk, that might be comparable to extreme events that might occur in the future.

The analysis further indicates the importance of morphological variables and trajectory of river longitudinal adjustment as compared to different drivers or receiver of changes. Sediment connectivity emerges as a potentially critical factor (Fig. 7, Fig. 8) [as highlighted by^62,89,96^], being connected to both changes in channel properties (HSF), and increasing decadal trends in flood hazard, independently from scaling (Fig. 7). The network and clustering analysis underlines how trends in flood events are ultimately governed by a balance of energy associated with atmosphere, flows of water combined with erosion, and transport.

## The Way Forward

Identify relationships between flows, catchment drivers, and downstream hydraulic geometry is currently complex. The current existing relationships are hard to compare exhaustively because they are derived from extensive field surveys or gaging stations that changed during the years, they are estimated using hydraulic scaling principles developed for larger regions, and they have been derived with varying flow levels in different studies, and through several different methods of fitting lines to relationships.

The proposed approach produces a robust and consistent estimate of longitudinal river adjustment across multiple watersheds. This describes how the dynamic properties of a river channel accommodate increases in discharge. Nevertheless, the capacity of a flood to modifying or being modified by channel properties is also strongly influenced by the availability of sediments in the landscape. With this in mind, *sediment data might not a*lways be available or be very hard to determine. This work shows how investigating the topography-based index of sediment connectivity allows providing additional information into the variability of flood hazard.

The HSF and sediment connectivity within this framework are derived from a Lidar capturing the landscape at one point in time, but the grouping of data indicates that neither general climatic setting nor the size of the drainage basin has any consistent influence on the downstream relations or the value of connectivity. HSFs do, however, seem to be related to changes in discharge and potential sediment load, and both parameters essentially correspond to an integral result of the long-term history of processes that have acted over the landscape. Overall, our findings suggest that climate or landscape alone do not explain changes in flood hazard. Rather, they act together with sediment connectivity and river network properties, fundamentally altering the frequency and magnitude of flood events.

The nature of non-linearity of the analysed relationships suggests that one should also consider that there is significant variability in the response of different geomorphic and extreme events across watersheds. While all considered watersheds will likely respond to extreme climatological events, they will react differently to the same magnitude of forcing, and the same geomorphic system may itself respond differently, depending on its condition at the time of the forcing.

Trends in flood hazard are often addressed considering climatic, hydrologic or geomorphological processes as independent flood drivers. Our results highlight that this is not enough to understand hazards that are intertwined with the complex dynamics of river systems and hillslope processes. Studying independent drivers alone might be insufficient and misleading for projecting flood risk over long timescales, especially when shifts in river morphology may be significant. Although it is widely acknowledged that flood change may be caused by several drivers that act at the same time, multidriver attribution studies are rare. In this paper we suggest a holistic view of flood risk, where we differentiate between drivers representing the three compartments potentially responsible for river flood change: atmosphere, landscape, and river conveyance. Our findings pinpoint the importance of considering river longitudinal variability and sediment properties in drawing flood trends, suggesting that these elements together with atmospheric and flow drivers, should be considered in modelling future scenarios and drawing associated management strategies.

## Material and Methods

**Figure 9:**
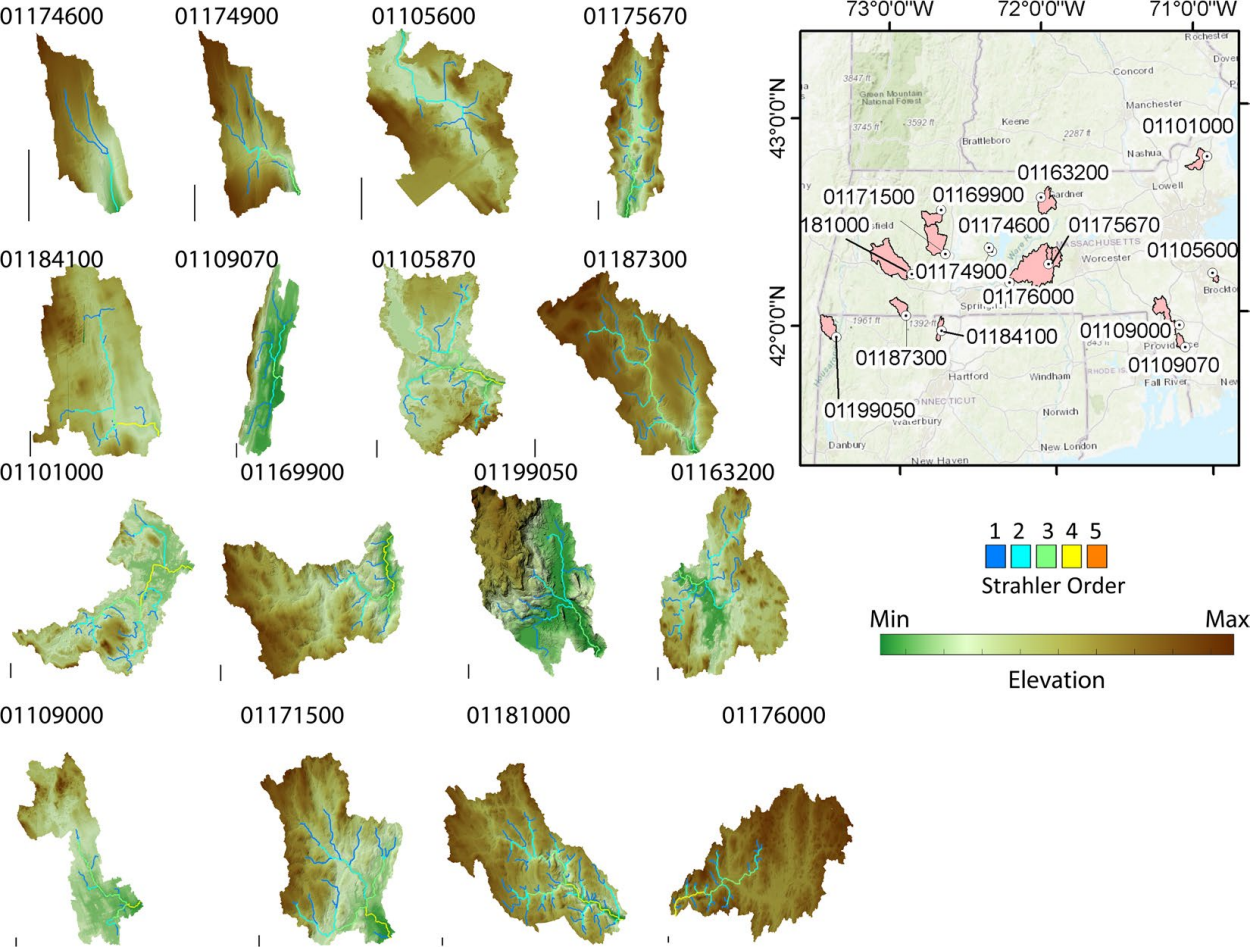
study areas and available lidar data, and drainage network with Strahler order. USGS Station ID is reported for each watershed, as described in dataset S1, S2 in the supplements. The bar near each watershed is scaled to 1 km. Acquisition dates for the Lidar range from 2010 to 2015 (for the sites in Massachusetts) and 2016 (for the sites in Connecticut). The declared vertical accuracy for the data range between 0.06 and 1.10 m. The figure has been arranged using Matlab 2018b [https://www.mathworks.com/release2018b].

The study considers sixteen watersheds in Connecticut and Massachusetts (USA, Fig. 9). The selected catchments belong to the physiographic division of the Appalachian Highland, and the physiographic province of New England. Most of them belongs to the New England Upland section (01187300, 01181000, 01171500, 01169900, 01174600, 01174900, 01163200, 01175670, 01184100, 01096000, 01170100), five sites belong to the Seaboard Lowland (01101000, 01105870, 01109070, 01109000, 01105600) and one to the Taconic section (01199050).

Available information for each watershed include (i) field surveyed bankfull width and drainage area at the outlet, bankfull discharge and its return period (***Dataset S1***, supplements, retrieved from^97^); (ii) daily discharge (Q) records for United States Geological Survey (USGS) gauging stations, (iii) Lidar high-resolution topography, in the form of Digital Terrain Models (DTMs) (Fig. 9).

Additionally, for each watershed, according to a geomorphologically extracted network^98^ (Supplement, Chapt 1.1), we defined drainage density D_d_, the watershed order Ω, drainage frequency (D_f_) and the length of the main stem of the network, where the main stem is defined as the network reach that at each junction drains the greatest portion of the watershed (***Dataset S1***, supplements). These parameters are often used to compare basins of different size and to establish catchment‐scale hydrological parameters^99^.

This chapter offers a brief description and an explanation of the rationale behind the choice of the various drivers. The reader should refer to the supplementary materials for a detailed description of the considered techniques.

### River geometry

Aspects of the river channel that relate to sediment, planform, and flow resistance are key to understanding conveyance capacity and overbank flooding potential^100^, as conveyance is reflected in the meander length and slope of the river channel^100^.

When trying to understand the linkage with flooding, an important parameter to focus on is the *bankfull hydraulic geometry*, which provides information on the channel’s morphology and storage capacity^40^. This concept emerges from the field evidence that rivers are in a perpetual state of flux and constantly adapt to recent floods and changing sediment loads^101^. The study of hydraulic geometry has been prominent starting from the 1960s and highlighted how channel capacity and widths scale with bankfull discharge, the latter typically being the discharge with a one to two-year recurrence interval^40,47,102–108^. While regime models based on a single flood discharge yield meaningful predictions of average conditions in many systems, formative flow and effective discharge diverge in some circumstances^101,109–111^, calling into question the veracity of the underlying assumption of equilibrium between a single discharge (however, it is defined) and the reach‐average channel dimensions. Differences across watersheds regarding flow regimes, catchment size, regional climatic and physiographic factors, geological characteristics, the responsiveness of the catchment, and human activities^40,47,102–108^ can be described observing adjustment of hydraulic geometry as power-law functions (Hydraulic Scaling Function HSF) relating bankfull properties and flows in the downstream direction along the river profile.

This paper focuses on HSF derived automatically from Lidar DTMs^112,113^ (Supplement, Chapt 1.2, Dataset S2). The proposed lidar-based algorithm uses a statistical approach to delineate threshold landscape curvatures^114–116^ for defining the hydrologic floodplain where the river can flow (overall valley shape)^98,112,113,117^, and thresholds of local curvature for defining homogeneous reaches along the river and to measure their bankfull width.

### Sediment connectivity

Among catchment characteristics, an important catchment driver that enhance our comprehension of landscape processes and water modeling is sediment connectivity^61^. As a proxy, we investigated the topography-based index of sediment connectivity (IC^42,118^) (Supplement, chapt 1.2). This index provides information about the percentage of the topographically defined catchment area that contributes to sediment runoff, and thus relates to the geomorphic effectiveness of floods. In particular, IC evaluates the potential connection between hillslopes and features (in this case the investigated channel), which act as targets for the potentially transported sediment. The main advantage of this index is that it considers impedance to sediment flows according to a weight factor that can be based on different methods. For a detailed analysis, we considered different parameters of the IC, including the mean, the 30 and 95 percentiles, the coefficient of variation and the percentage of areas with different degrees of connectivity (Dataset S2 in the supplement).

### Flows

Understanding flow signatures as they relate to catchment properties summarize river flow dynamics^119^, offering a way to understand flood generation processes and hence their flood frequency response^43^. Different parts of the flow spectrum have different degrees of influence on hydro-geomorphic processes. High-magnitude low-frequency flows, associated with overbank flows and thus flood hazard are often relevant to significant and abrupt geomorphic changes. Low-magnitude flows determine habitat conditions for the survival and the functioning of indigenous riverine biota and therefore are relevant to long-term changes in eco-hydrology of river systems; and intermediate events which, being formative events, conduct most of the geomorphic work and are in dynamic equilibrium with the current morphological and geometric configuration of a given channel reach. Flow signatures can be identified by several different properties of the river flow^120^. In this work we focus primarily on characteristic properties of the daily flow distribution. Specifically, to characterize hydrologic flow regime in each catchment, we estimated the low (30^th^), high (95^th^), average and coefficient of variation of discharge and the bankfull discharge estimates. To permit comparison among catchments of different drainage area, we expressed flow quantities as specific discharge (i.e. flow normalized by catchment area). The flow data were retrieved from all current and discontinuous water data for the study sites from the USGS National Water Information System (NWIS) and corresponded to various record lengths but with more than 30 yrs of record for all stations (Dataset S2 in the supplement).

### Climate (Rainfall)

*Precipitation* is one of the most significant water cycle components and the primary driver for floods. Any changes in precipitation (as direct water input) and temperature (as controlling factor of snowmelt and evapotranspiration) impacts the magnitude-frequency of water discharges entering a channel reach, possibly leaving a signature on river properties. Precipitation concentration is an important parameter of the climatic description of a single location or region, which complements the information provided by other, more common, variables such as the annual precipitation or the seasonality^121^. The CI (Concentration Index)^122^ is able to relate the magnitude of the precipitation events to the time period in which they occur. The CI can act as an estimator of rainfall erosivity^123^ as well as of the repercussions of climate change on water cycle^124^ and floods^78^. For this reason, the analysis of daily precipitation concentration is an important aspect to consider in order to evaluate both hydrological and geomorphological drivers linked to flood hazard. For this work, we will consider the CI values published by^125^ (Dataset S1 in the supplement) to characterize the climatology of rainfall intensity regime in our study areas and will thus refer to CI when we mention “Climate”, and we refer to a climatologic characteristics of precipitation.

### Trends on flow exceedance

We considered trends for two distinct flow thresholds representing low flows (Q_30_) and high flows (Q_95_). For each site, we estimated the Q_30_ and Q_95_ values and counted for each year the total number of events exceeding the considered threshold for at least 5 consecutive days. Trends in flow exceeding Q_30_ are considered as a proxy of changes in low flow (baseflow) regime, while trends of events exceeding the Q_95_ thresholds are considered in this study as a proxy of trends in “flood hazard”, acknowledging, however, that the events identified in this way do not necessarily lead to water outside the river banks (i.e. flood inundation).

For identification of temporal trends in flow exceedance and their significance, we applied a mean unbiased exponential least squares curve^38^, which allowed us to avoid predicting negative values.

### Connectivity and flow distribution analysis

Statistical analysis of connectivity and flow properties is carried out to identify similarities and differences and classify hydrologic regimes and landscapes across the analyzed watersheds. When one wants to estimate the probability of a flood and develop suitable means to mitigate its effects, one needs to determine a threshold value and calculate the probability that the river discharge data do not exceed the chosen threshold value^126^ or equivalently estimate the probability that the threshold will be exceeded^127^. Similarly, to identify fully disconnected landscape, one has to estimate the probability of a connectivity value, determine a threshold value (i.e^17,60^) and calculate the probability that the IC does not exceed the chosen threshold value.

In statistics, the probability of non-exceedance is expressed based on the cumulative distribution function (CDF), while equivalently the probability of exceedance is expressed using the complementary CDF (or cCDF), which is also termed “flow duration curve” in hydrology^44^. In this work we present both CDF and cCDF because they allow us to visually emphasize the left and right hand tails (i.e. extremes) of the distributions.

### Interdependence of variables

Recognizing the potential impact of outliers in deriving relationships based on a relatively small sample size, we emphasize that this study is not meant to develop a general model, but rather to carry out a forensic investigation and document the possible existence of a relationship among the different factors examined. On the long run, this work will offer a starting point to aggregating enough studies to provide a clearer understanding of the complex interactions under analysis. In the process of changing the hydro-geomorphological organization, systems traverse various phases, following (possibly) non-linear rules. To investigate the relationships between variables, we used the distance correlation index, dCor^128^. This index has the advantage of being able to capture non-monotonic associations and ensure that the correlation is zero if and only if the variables are statistically independent, which is not the case for, e.g. Pearson’s product-moment correlation. The range of dCor is 0 to 1, and the closer dCor is to 1, the stronger the dependence between the variables. The statistical significance was assessed using the distance covariance test with 1000 replicates^128^, and significance was tested at the 5, 10 and 25% significance level.

The distance correlation has been then further used as a measure of dissimilarity (1-dCorr) to identify a hierarchical clustering of the different variables^129^. This analysis has been done to identify groups of variables mostly connected, rather than to define a model for the variables interaction.

The same dissimilarity measure has been used to create a undirected network^130^ to represent the system of interacting units in the hydrological and geomorphological realm. The network structure can visually formulate nonlinearity, solely based on the considered data without requiring knowledge about the underlying physical processes. The network has been characterized by some centrality measures, named *Betweenness* (the number of shortest paths that the focal node lies on), *Closeness* (the mean shortest path between a focal node and all other nodes in the network), *Degree* (the number of edges that connect the focal node to other nodes), and *Strength* (measures the strength of vertices in terms of the total weight of their connections).

## Data and software availability

The considered Lidar data and discharge data are of public domain from the open web-services of^131^ and^132^. The climatic data were provided by^125^ and are freely available on GitHub. The analysis of sediment connectivity was carried out using the open-source codes provided on GitHub by^133^. The correlation analysis and clustering was performed in R using the package by^134^. Network analysis was also implemented in R using the package^135^. KDE in Fig. 7 has been evaluated using the R package by^136^ and interpolated using^137^. Flow trends have been evaluated considering the work of^38^ and the codes available on GitHub (https://github.com/LouiseJSlater/GRL2015). The codes for the network extraction and the HSF computation from lidar are available upon request to the main author of this paper.

## Supplementary information

Supplementary information.

## References

[1] Roder G (2017). Assessment of social vulnerability to floods in the floodplain of Northern Italy. Wea. Climate Soc..

[2] Hallegatte S, Green C, Nicholls RJ, Corfee-Morlot J (2013). Future flood losses in major coastal cities. Nat. Clim. Chang..

[3] Wohl, E. E. *Inland Flood Hazards: Human, Riparian, and Aquatic Communities*. (Cambridge University Press, 2000).

[4] Blöschl G (2019). Changing climate both increases and decreases European river floods. Nature.

[5] Mallakpour I, Villarini G (2015). The changing nature of flooding across the central United States. Nat. Clim. Chang..

[6] Dottori F (2018). Increased human and economic losses from river flooding with anthropogenic warming. Nat. Clim. Chang..

[7] Winsemius HC (2016). Global drivers of future river flood risk. Nat. Clim. Chang..

[8] Arnell NW, Gosling SN (2016). The impacts of climate change on river flood risk at the global scale. Clim. Change.

[9] Montanari A, Koutsoyiannis D (2014). Modeling and mitigating natural hazards: Stationarity is immortal!. Water Resour. Res..

[10] Koutsoyiannis D, Montanari A (2015). Negligent killing of scientific concepts: the stationarity case. Hydrol. Sci. J..

[11] Milly PCD, Dunne KA, Vecchia AV (2005). Global pattern of trends in streamflow and water availability in a changing climate. Nature.

[12] Milly PCD (2015). On critiques of ‘Stationarity is dead: Whither water management?’. Water Resour. Res..

[13] Blöschl G (2017). Changing climate shifts timing of European floods. Science.

[14] Viglione A (2016). Attribution of regional flood changes based on scaling fingerprints. Water Resour. Res..

[15] Wohl Ellen (2019). Forgotten Legacies: Understanding and Mitigating Historical Human Alterations of River Corridors. Water Resources Research.

[16] Tarolli P, Cao W, Sofia G, Evans D, Ellis EC (2019). From features to fingerprints: A general diagnostic framework for anthropogenic geomorphology. Progress in Physical Geography: Earth and Environment.

[17] Tarolli P, Sofia G (2016). Human topographic signatures and derived geomorphic processes across landscapes. Geomorphology.

[18] Thompson SE (2013). Developing predictive insight into changing water systems: use-inspired hydrologic science for the Anthropocene. Hydrol. Earth Syst. Sci..

[19] Blum MD, Roberts HH (2009). Drowning of the Mississippi Delta due to insufficient sediment supply and global sea-level rise. Nat. Geosci..

[20] Degu Ahmed Mohamed, Hossain Faisal, Niyogi Dev, Pielke Roger, Shepherd J. Marshall, Voisin Nathalie, Chronis Themis (2011). The influence of large dams on surrounding climate and precipitation patterns. Geophysical Research Letters.

[21] Santer, B. D. *et al*. Human influence on the seasonal cycle of tropospheric temperature. *Science* **361** (2018). 10.1126/science.aas8806 30026201

[22] Dingle EH (2019). Decadal-scale morphological adjustment of a lowland tropical river. Geomorphology.

[23] Pfeiffer Allison M., Collins Brian D., Anderson Scott W., Montgomery David R., Istanbulluoglu Erkan (2019). River Bed Elevation Variability Reflects Sediment Supply, Rather Than Peak Flows, in the Uplands of Washington State. Water Resources Research.

[24] Sholtes JS, Yochum SE, Scott JA, Bledsoe BP (2018). Longitudinal variability of geomorphic response to floods. Earth Surf. Processes Landforms.

[25] Surian N (2016). Channel response to extreme floods: Insights on controlling factors from six mountain rivers in northern Apennines, Italy. Geomorphology.

[26] Scorpio V (2018). Basin-scale analysis of the geomorphic effectiveness of flash floods: A study in the northern Apennines (Italy). Sci. Total Environ..

[27] Guan M, Carrivick JL, Wright NG, Sleigh PA, Staines KEH (2016). Quantifying the combined effects of multiple extreme floods on river channel geometry and on flood hazards. J. Hydrol..

[28] Munoz SE (2018). Climatic control of Mississippi River flood hazard amplified by river engineering. Nature.

[29] Sofia G (2019). On the linkage between runoff generation, land drainage, soil properties, and temporal patterns of precipitation in agricultural floodplains. Adv. Water Resour..

[30] Zhang S, Guo Y, Wang Z (2015). Correlation between flood frequency and geomorphologic complexity of rivers network - A case study of hangzhou China. J. Hydrol..

[31] Criss RE, Shock EL (2001). Flood enhancement through flood control. Geology.

[32] Lane SN, Tayefi V, Reid SC, Yu D, Hardy RJ (2007). Interactions between sediment delivery, channel change, climate change and flood risk in a temperate upland environment. Earth Surf. Processes Landforms.

[33] Neuhold, C., Stanzel, P. & Nachtnebel, H. P. Incorporating river morphological changes to flood risk assessment: uncertainties, methodology and application. vol. 9 789–799 (2009).

[34] Slater, L. J., Khouakhi, A. & Wilby, R. L. River channel conveyance capacity adjusts to modes of climate variability. *Sci. Rep*. (2019). 10.1038/s41598-019-48782-1 PMC6718627 31477746

[35] Neri A, Villarini G, Slater LJ, Napolitano F (2019). On the statistical attribution of the frequency of flood events across the U.S. Midwest. Advances in Water Resources.

[36] Fryirs K, Lisenby P, Croke J (2015). Morphological and historical resilience to catastrophic flooding: The case of Lockyer Creek, SE Queensland, Australia. Geomorphology.

[37] Grill G (2019). Mapping the world’s free-flowing rivers. Nature.

[38] Slater LJ, Singer MB, Kirchner JW (2015). Hydrologic versus geomorphic drivers of trends in flood hazard. Geophys. Res. Lett..

[39] Bracken LJ, Wainwright J (2006). Geomorphological Equilibrium: Myth and Metaphor?. Transactions of the Institute of British Geographers.

[40] Julien P. Y. (2015). Downstream hydraulic geometry of alluvial rivers. Proceedings of the International Association of Hydrological Sciences.

[41] Heckmann T (2018). Indices of sediment connectivity: opportunities, challenges and limitations. Earth-Sci. Rev..

[42] Cavalli M, Trevisani S, Comiti F, Marchi L (2013). Geomorphometric assessment of spatial sediment connectivity in small Alpine catchments. Geomorphology.

[43] Donnelly C, Andersson JCM, Arheimer B (2016). Using flow signatures and catchment similarities to evaluate the E-HYPE multi-basin model across. Europe. Hydrol. Sci. J..

[44] Castellarin, A., Vogel, R. M. & Brath, A. A stochastic index flow model of flow duration curves. *Water Resources Research* **40** (2004).

[45] Wohl E, Merritt DM (2008). Reach-scale channel geometry of mountain streams. Geomorphology.

[46] Ferguson RI (1986). Hydraulics and hydraulic geometry. Progress in Physical Geography: Earth and Environment.

[47] Gleason CJ (2015). Hydraulic geometry of natural rivers: A review and future directions. Prog. Phys. Geogr..

[48] Bieger, K., Rathjens, H., Allen, P. M. & Arnold, J. G. Development and Evaluation of Bankfull Hydraulic Geometry Relationships for the Physiographic Regions of the United States. **51**, 842–858 (2015).

[49] Faustini JM, Kaufmann PR, Herlihy AT (2009). Downstream variation in bankfull width of wadeable streams across the conterminous United States. Geomorphology.

[50] Castro JM, Jackson PL (2001). Bankfull discharge recurrence intervals and regional hydraulic geometry relationships: patterns in the Pacific Northwest, USA. J. Am. Water Resour. Assoc..

[51] Doll BA (2002). Hydraulic geometry relationships for urban streams through the Piedemont of North Carolina. JAWRA Journal of the American Water Resources Association.

[52] Golden LA, Springer GS (2006). Channel geometry, median grain size, and stream power in small mountain streams. Geomorphology.

[53] Hession WC, Pizzuto JE, Johnson TE, Horwitz RJ (2003). Influence of bank vegetation on channel morphology in rural and urban watersheds. Geology.

[54] Moody, T., Wirtanen, M. & Yard, S. N. Regional relationships for bankfull stage in natural channels of the arid southwest. Natural Channel Design Inc.*, Flagstaff* (2003).

[55] Gleason CJ, Smith LC (2014). Toward global mapping of river discharge using satellite images and at-many-stations hydraulic geometry. Proceedings of the National Academy of Sciences.

[56] Galster JC (2007). Natural and anthropogenic influences on the scaling of discharge with drainage area for multiple watersheds. Geosphere.

[57] Park CC (1978). Allometric analysis and stream channel morphometry. Geogr. Anal..

[58] Johnson PA, Heil TM (1996). Uncertainty in estimating bankfull conditions. JAWRA Journal of the American Water Resources Association.

[59] Parsons, A. J., Wainwright, J. & Brazier, R. E. Is sediment delivery a fallacy? *and Landforms: The …* (2006).

[60] Calsamiglia A (2018). Effects of agricultural drainage systems on sediment connectivity in a small Mediterranean lowland catchment. Geomorphology.

[61] Wohl E (2019). Connectivity as an emergent property of geomorphic systems. Earth Surf. Processes Landforms.

[62] Croke J, Fryirs K, Thompson C (2013). Channel–floodplain connectivity during an extreme flood event: implications for sediment erosion, deposition, and delivery. Earth Surf. Processes Landforms.

[63] Nones M (2019). Dealing with sediment transport in flood risk management. Acta Geophysica.

[64] Amrhein V, Greenland S, McShane B (2019). Scientists rise up against statistical significance. Nature.

[65] Döll P, Schmied HM (2012). How is the impact of climate change on river flow regimes related to the impact on mean annual runoff? A global-scale analysis. Environ. Res. Lett..

[66] Falloon PD, Betts RA (2006). The impact of climate change on global river flow in HadGEM1 simulations. Atmos. Sci. Lett..

[67] Hodgkins GA (2017). Climate-driven variability in the occurrence of major floods across North America and Europe. J. Hydrol..

[68] Novotny EV, Stefan HG (2007). Stream flow in Minnesota: Indicator of climate change. J. Hydrol..

[69] Masselink RJH (2016). Modelling discharge and sediment yield at catchment scale using connectivity components. Land Degrad. Dev..

[70] Hack JT (1957). Studies of longitudinal stream profiles in Virginia and Maryland. US Geological Survey Professional Paper.

[71] Reis A. Heitor (2006). Constructal view of scaling laws of river basins. Geomorphology.

[72] Horton R (1932). ~E. Drainage basin characteristics. Eos Trans. Amer. Geophys. Union.

[73] Hunt AG (2016). Brief communication: Possible explanation of the values of Hack’s drainage basin, river length scaling exponent. Nonlinear Process. Geophys..

[74] Tarboton DG, Bras RL, Rodriguez-Iturbe I (1988). The fractal nature of river networks. Water Resour. Res..

[75] Rodríguez-Iturbe, I. & Rinaldo, A. *Fractal River Basins: Chance and Self-Organization*. (Cambridge University Press, 2001).

[76] Phillips JD (2012). Networks of Historical Contingency in Earth Surface Systems. J. Geol..

[77] Ruiz-Villanueva V (2018). Impacts of a large flood along a mountain river basin: Unravelling the geomorphic response and large wood budget in the upper Emme River (Switzerland). Earth Surface Dynamics Discuss.

[78] Sofia, G., Roder, G., Dalla Fontana, G. & Tarolli, P. Flood dynamics in urbanised landscapes: 100 years of climate and humans’ interaction. *Sci. Rep*. **7**, (2017). 10.1038/srep40527 PMC5228191 28079147

[79] Kundzewicz ZW (2014). Flood risk and climate change: global and regional perspectives. Hydrol. Sci. J..

[80] Kron, W., Eichner, J. & Kundzewicz, Z. W. Reduction of flood risk in Europe–Reflections from a reinsurance perspective. *J. Hydrol*. (2019).

[81] Pallard B, Castellarin A, Montanari A (2009). A look at the links between drainage density and flood statistics. Hydrol. Earth Syst. Sci..

[82] Milly PCD (2008). Stationarity Is Dead: Whither Water Management?. Science.

[83] Kundzewicz ZW (2018). Uncertainty in climate change impacts on water resources. Environ. Sci. Policy.

[84] Heckmann T, Schwanghart W (2013). Geomorphic coupling and sediment connectivity in an alpine catchment — Exploring sediment cascades using graph theory. Geomorphology.

[85] Keesstra S (2018). The way forward: Can connectivity be useful to design better measuring and modelling schemes for water and sediment dynamics?. Sci. Total Environ..

[86] Boardman, J. & Poesen, J. Soil Erosion in Europe: Major Processes, Causes and Consequences. in *Soil Erosion in Europe* 477–487 (John Wiley & Sons, Ltd, 2006).

[87] Prosdocimi M (2017). Rainfall simulation and Structure-from-Motion photogrammetry for the analysis of soil water erosion in Mediterranean vineyards. Sci. Total Environ..

[88] Tarolli P, Cavalli M, Masin R (2019). High-resolution morphologic characterization of conservation agriculture. Catena.

[89] Kalantari Z (2019). Assessing flood probability for transportation infrastructure based on catchment characteristics, sediment connectivity and remotely sensed soil moisture. Sci. Total Environ..

[90] Chen S-A, Michaelides K, Grieve SWD, Singer MB (2019). Aridity is expressed in river topography globally. Nature.

[91] Stark CP (2010). The climatic signature of incised river meanders. Science.

[92] Ceola S, Laio F, Montanari A (2019). Global-scale human pressure evolution imprints on sustainability of river systems. Hydrol. Earth Syst. Sci..

[93] Andrews, E. D. & Nankervis, J. M. Effective discharge and the design of channel maintenance flows for gravel-bed rivers. in 151–164 (American Geophysical Union (AGU), 1995).

[94] Hooke JM (2015). Variations in flood magnitude–effect relations and the implications for flood risk assessment and river management. Geomorphology.

[95] Wicherski W, Dethier DP, Ouimet WB (2017). Erosion and channel changes due to extreme flooding in the Fourmile Creek catchment, Colorado. Geomorphology.

[96] Bracken LJ, Croke J (2007). The concept of hydrological connectivity and its contribution to understanding runoff-dominated geomorphic systems. Hydrol. Process..

[97] Bent, G. C. G. C. & Waite, A. M. A. M. *Equations for Estimating Bankfull Channel Geometry and Discharge for Streams in Massachusetts*. 62pp (2013).

[98] Sofia G., Tarolli P., Cazorzi F., Dalla Fontana G. (2011). An objective approach for feature extraction: distribution analysis and statistical descriptors for scale choice and channel network identification. Hydrology and Earth System Sciences.

[99] Mutzner R, Tarolli P, Sofia G, Parlange MB, Rinaldo A (2016). Field study on drainage densities and rescaled width functions in a high-altitude alpine catchment. Hydrol. Process..

[100] Bjerklie DM (2007). Estimating the bankfull velocity and discharge for rivers using remotely sensed river morphology information. J. Hydrol..

[101] Davidson SL, Eaton BC (2018). Beyond Regime: A Stochastic Model of Floods, Bank Erosion, and Channel Migration. Water Resour. Res..

[102] Singh, V. P., Yang, C. T. & Deng, Z. Q. Downstream hydraulic geometry relations: 1. Theoretical development. *Water Resour. Res*. **39**, (2003).

[103] Singh, V. P., Yang, C. T. & Deng, Z.-Q. Downstream hydraulic geometry relations: 2. Calibration and testing. *Water Resources Research* vol. 39 (2003).

[104] Leopold L. B. & Maddock, T. Jr. The hydraulic geometry of stream channels and some physiographic implications. **57** (1953).

[105] Wolman, M. G. & Leopold, L. B. River flood plains: some observations on their formation. vols **282-C** (1957).

[106] Leopold L. B. A View of the River. (Harvard University Press, Cambridge, Massachussetts, 1994).

[107] Leopold L. B., Wolman, M. G. & Miller, J. P. *Fluvial processes in geomorphology, 522 pp*. 522 (San Francisco, Freeman, 1964).

[108] Wohl E (2004). Limits of downstream hydraulic geometry. Geology.

[109] Doyle MW, Shields D, Boyd KF, Skidmore PB, Dominick D (2007). Channel-forming discharge selection in river restoration design. J. Hydraul. Eng..

[110] Lenzi MA (2001). Step-pool evolution in the Rio Cordon, Northeastern Italy. Earth Surf. Processes Landforms.

[111] Pickup G, Warner RF (1976). Effects of hydrologic regime on magnitude and frequency of dominant discharge. J. Hydrol..

[112] Sofia G, Tarolli P, Cazorzi F, Dalla Fontana G (2015). Downstream hydraulic geometry relationships: Gathering reference reach-scale width values from LiDAR. Geomorphology.

[113] Sofia G, Di Stefano C, Ferro V, Tarolli P (2017). Morphological Similarity of Channels: From Linear Erosional Features (Rill, Gully) to Alpine Rivers. Land Degrad. Dev..

[114] Gorini, M. A. V. & Abelha Mota, G. L. Which is the best scale? Finding fundamental features and scales in DEMs. In *Geomorphometry 2011* (eds. Hengl, T., Evans, I. S, Wilson, J. P & Gould, M.) 67–70 (2011).

[115] Wood, J. D. The geomorphological characterisation of digital elevation models. (University of Leicester, UK, 1999).

[116] Evans, I. S., Young, M. & Gill, J. S. An integrated system of terrain analysis and slope mapping, final report. *Univ. of Durham, Durham, NC* (1979).

[117] Lo Re Giulia, Fuller Ian C., Sofia Giulia, Tarolli Paolo (2018). High-resolution mapping of Manawatu palaeochannels. New Zealand Geographer.

[118] Borselli L, Cassi P, Torri D (2008). Prolegomena to sediment and flow connectivity in the landscape: A GIS and field numerical assessment. Catena.

[119] Blöschl, G., Sivapalan, M., Wagener, T., Savenije, H. & Viglione, A. *Runoff Prediction in Ungauged Basins: Synthesis Across Processes, Places and Scales*. (Cambridge University Press, 2013).

[120] Kuentz, A., Arheimer, B. & Hundecha, Y. Understanding hydrologic variability across Europe through catchment classification. *Hydrol. Earth Syst. Sci*. (2017).

[121] Serrano-Notivoli R (2018). Spatio-temporal variability of daily precipitation concentration in Spain based on a high-resolution gridded data set. Int. J. Climatol..

[122] Martin-Vide J (2004). Spatial distribution of a daily precipitation concentration index in peninsular Spain. Int. J. Climatol..

[123] de Luis M, Brunetti M, Gonzalez-Hidalgo JC, Longares LA, Martin-Vide J (2010). Changes in seasonal precipitation in the Iberian Peninsula during 1946–2005. Glob. Planet. Change.

[124] Vicente-Serrano SM (2006). Differences in Spatial Patterns of Drought on Different Time Scales: An Analysis of the Iberian Peninsula. Water Resour. Manage..

[125] Royé D, Martin-Vide J (2017). Concentration of daily precipitation in the contiguous United States. Atmos. Res..

[126] Bowers MC, Tung WW, Gao JB (2012). On the distributions of seasonal river flows: Lognormal or power law?. Water Resour. Res..

[127] Fernandes W, Naghettini M, Loschi R (2010). A Bayesian approach for estimating extreme flood probabilities with upper-bounded distribution functions. Stochastic Environmental Research and Risk Assessment.

[128] Székely GJ, Rizzo ML, Bakirov NK (2007). Measuring and testing dependence by correlation of distances. Ann. Stat..

[129] Szekely GJ, Rizzo ML (2005). Hierarchical Clustering via Joint Between-Within Distances: Extending Ward’s Minimum Variance Method. J. Classification.

[130] Phillips JD, Schwanghart W, Heckmann T (2015). Graph theory in the geosciences. Earth-Sci. Rev..

[131] CtECO, C. E. C. O. Connecticut Elevation (Lidar) Data., http://www.cteco.uconn.edu/data/lidar/index.htm (2015).

[132] Massachusetts Document Repository. https://docs.digital.mass.gov/dataset/massgis-data-lidar-terrain-data.

[133] Crema, S., Marchi, L. & Cavalli, M. SedInConnect: free and stand-alone software for assessing sediment connectivity. 10.13140/RG.2.2.34402.58569 (2019).

[134] Rizzo, M. L. & Szekely, G. J. Energy: E-Statistics: Multivariate Inference via the Energy of Data (R Package), Version 1.7-0. (2017).

[135] CRAN - Package igraph. https://CRAN.R-project.org/package=igraph.

[136] Le Bourlot, V. R Package STDiag. https://rdrr.io/rforge/STdiag/

[137] CRAN- Package akima (1974). Akima, H. A Method of Bivariate Interpolation and Smooth Surface Fitting Based on Local Procedures. CACM.

[138] Venables W. N., Ripley B. D. (2002). Modern Applied Statistics with S.

[139] Martz LW, Garbrecht J (1992). Numerical definition of drainage network and subcatchment areas from Digital Elevation Models. Comput. Geosci..

[140] Thwaites FT (1939). Physiography of Eastern United States. Nevin M. Fenneman. The Journal of Geology.

[141] Fenneman NM (1946). Physiographic Divisions of the United States. Annals of the Association of American Geographers.

[142] Crema S, Schenato L, Goldin B, Marchi L, Cavalli M (2015). Toward the development of a stand-alone application for the assessment of sediment connectivity. Rendiconti online della Società Geologica Italiana.

