# Supplementary material for: Floods and rivers: a circular causality perspective
Source: Sci Rep. 2020 Mar 20;10:5175. doi: 10.1038/s41598-020-61533-x (PMC7083824; doi:10.1038/s41598-020-61533-x)
Supplement: Supplementary file 1 — Supplementary information. [file 41598_2020_61533_MOESM1_ESM.docx]

**Floods and rivers: A circular causality perspective**

G.Sofia ^1*^

E. I. Nikolopoulos^2^

^1*^ [giulia.sofia@uconn.edu](mailto:giulia.sofia@uconn.edu), University of Connecticut, Department of Civil & Environmental Engineering, 261 Glenbrook Rd, Storrs, CT 06269

^2^ Florida Institute of Technology, Department of Mechanical and Civil Engineering, Florida Institute of Technology, Melbourne, FL 32901

**Contents of this file**

***Methods, including Figures***: detailed Methods used to prepare the data in the manuscript, Figures S1 to S4 and their captions and supporting references for the Methods

***Dataset S1, S2***: complete data used in the manuscript.

1. **Methods**
   1. ***Channel extraction***

This paper defines drainage networks identified according to [^1^](https://paperpile.com/c/kZI1KG/T5za), also considering that this type of channel network delineation plays an essential role in correctly capturing streamflow generation mechanism [^2^](https://paperpile.com/c/kZI1KG/iRYE).

The approach delineates channels by statistical thresholding of two landscape parameters: minimum curvature [^3^](https://paperpile.com/c/kZI1KG/MLm9) and topographic openness [^4^](https://paperpile.com/c/kZI1KG/KNS4). The coupling of the two indices avoids uncertainties related to the second derivative operation, and it can compensate for bias due to the different source of the considered Lidar DTMs.

The two indices are evaluated at various window sizes for a constant DTM resolution until an optimum scale is reached (fitting-enforcing approach [^1,5–7^](https://paperpile.com/c/kZI1KG/IGBm+T5za+8pC4+FKj8)). This optimum is automatically set as the scale (window or kernel) where the investigated parameter becomes most extreme (largest skewness).

After setting the optimum scale, the network extraction procedure follows four steps:

1. normalisation and overlapping of openness and curvature in order to highlight the most likely surface convergence,
2. identification of a weighted upslope area[^8^](https://paperpile.com/c/kZI1KG/RXwB), that defines flow paths and accumulation consistent with terrain geometry, where the contribution at each grid cell is taken as the grid cell area-weighted according to the likely surface convergence identified in (i).
3. identification of the channel network location where the z-score of the weighted upslope area is higher than 0[^1^](https://paperpile.com/c/kZI1KG/T5za).
4. production of a fully connected network through noise‐filtering and shortest-path analysis[^1^](https://paperpile.com/c/kZI1KG/T5za).

For each watershed, according to the geomorphologically extracted network, we further defined drainage density D_d_, the watershed order Ω, drainage frequency (D_f_), the length of the longest element of the network, and the length of the main stem of the network (***Dataset S1***). We define the longest element by subsequently tracing the maximum downstream distance in the upstream direction, and identify the main stem by subsequently tracing the maximum upslope area in the upstream direction.

- 1. ***Hydraulic Scaling Function (HSF)***

The paper will investigate HSF (Hydraulic Scaling Functions) relating downstream variations in bankfull widths (w_bkf_) to contributing area (A) ($w_{bkf}=\alpha A^{\beta}$ where α –scaling- and β –exponent- are constants [^9–17^](https://paperpile.com/c/kZI1KG/pSQIs+HlaY6+EEQDx+gRztm+yKyNc+YzTOx+D9ePp+uBtDO+eo2Tp) ) along a channel, in the longitudinal downstream direction [Dataset S2].

The advantage of using drainage area is that it is a parameter directly inferable from topography, without requiring an a-priori knowledge of the processes. While it might be a less reliable predictor of bankfull channel dimensions than discharge [^18^](https://paperpile.com/c/kZI1KG/tqJH), substituting drainage area for discharge is a reasonable first-order estimate to a wide range of geologic, hydrologic, and engineering approaches to watershed management [^19^](https://paperpile.com/c/kZI1KG/tpJd), and this practice has been used consistently to highlight differences in watershed characteristics, in terms of human impact, topography, sediment transport and climate (e.g. [^9–17^](https://paperpile.com/c/kZI1KG/pSQIs+HlaY6+EEQDx+gRztm+yKyNc+YzTOx+D9ePp+uBtDO+eo2Tp)).

The HSF analysis focuses on the main stem of the network (as it is the channel that tends to have larger volumes of runoff and debris as it collects from the smaller waterways flowing into them).

The technique to extract HSF automatically from Lidar has been already successfully applied in other parts of the world [^6,7^](https://paperpile.com/c/kZI1KG/8pC4+FKj8). The proposed method gives an average characterisation of the channel geometry (reach-scale width), rather than an exact one at each location of the network.

The core of the method is the evaluation of surface curvature [^3^](https://paperpile.com/c/kZI1KG/MLm9) which emphasises the concavities of a landscape. The extraction procedure comprises three steps:

1. definition of homogeneous reaches, and hydrologic floodplain;
2. characterisation of transect orthogonal to the thalweg;
3. statistical definition of the transect width, and aggregation to reach-scale width.

By definition, a homogeneous reach includes locations along the channel thalweg where the drainage area constantly increases, as sudden changes in the contributing area are related to lateral intakes from secondary tributaries.

The hydrologic floodplain defines the overall valley shape (total bankfull capacity), where the active channel can flow, and it encompasses the optimum scale of analysis defined using the fitting-enforcing approach for the whole watershed (Chapt 1.1).

Once the floodplain size is selected, the fitting-enforcing is re-computed for each homogeneous reach, assessing the skewness of the minimum curvature values within the floodplain, to define a reach-scale optimum window.

For each homogeneous reach, minimum curvature is computed with the reach-scale optimum kernel. A statistic threshold (where curvature deviates from normality [^6,20,21^](https://paperpile.com/c/kZI1KG/8pC4+JpSB+Fub9)) extracts localised widths on cross-sections orthogonal to the thalweg, as the width flanked by the points of intersection between the threshold line, and the curvature profile along the cross-section.

The bankfull width, referred to hereinafter, is the average value of widths extracted in each homogeneous reach.

The definition of river geometry from DTMs requires some underlying assumptions. On the digital model, the visible topographic river banks at each location reflect as the minimum width of the channel occupied by water at the moment of the survey. The channel geometry, furthermore, is approximated, depending on the DTM vertical quality, as well as on the depth of water at the time of the survey [^22^](https://paperpile.com/c/kZI1KG/pZ5x). This approximation can, however, correctly depict >70% of the total cross-section, with better performances, the higher the resolution of the DTM and the steeper the topography. The applied technique allows for a correct bankfull approximation because bankfull width is derived from curvature (Fig. S1), rather than from elevation directly. By this approach, we can account for the ‘flatness’ of Lidar elevation within channels occupied by water.

The example in Fig. S1 shows a profile of a river in Connecticut (USGS station 01209700) taken from Lidar, and the curvature evaluated on the same profile. For this station, the declared bankfull width from USGS surveys is 19.5 m, extracted bankfull width is 19.3 m.


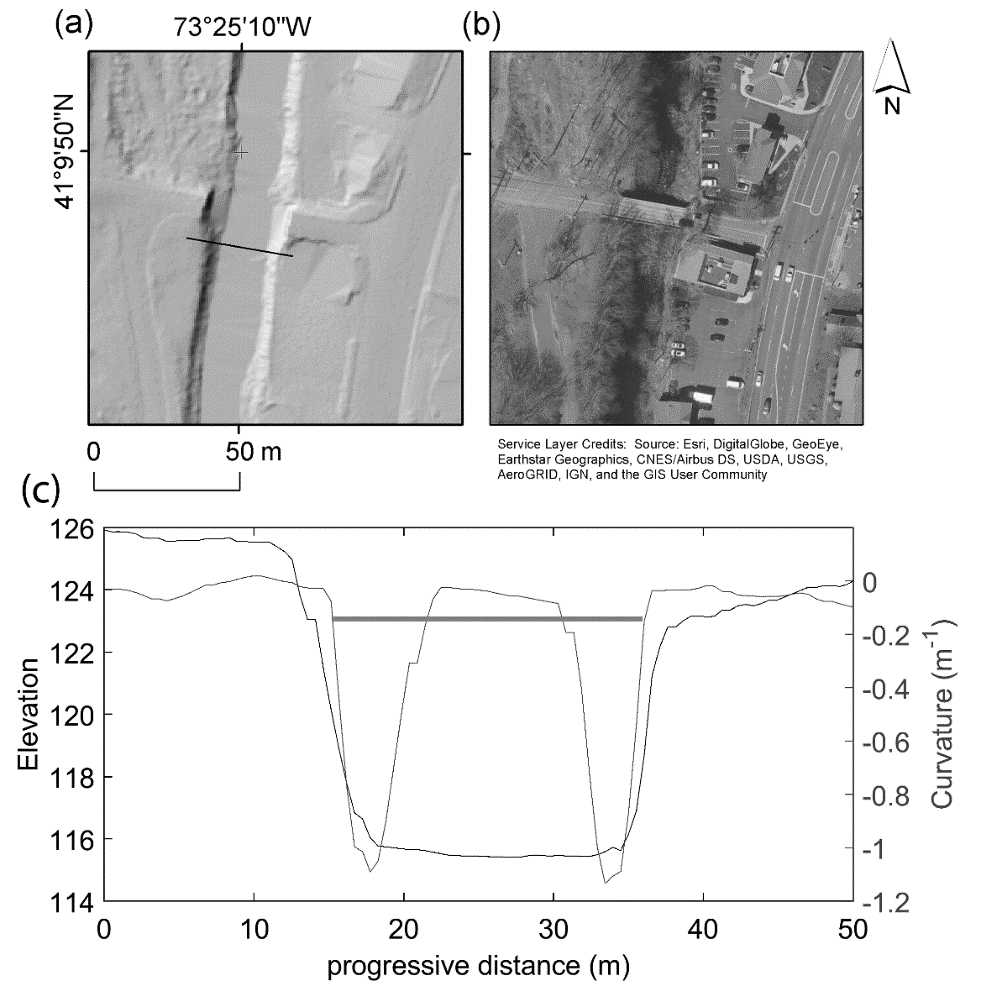


Figure S1 Elevation and curvature evaluated for the USGS station 01209700. Declared bankfull width from USGS surveys is 19.5 m, extracted bankfull width (red line) is 19.33 m. The images in (a) and (b) have been arranged using ArcGis 10.7 [www.arcgis.com]

The HSF derived using the con (Dataset S2) show a good agreement (R^2^ always statistically significant and on average >0.6) with those reported in [^23^](https://paperpile.com/c/kZI1KG/EEkW), and an RMSE in the same order of the DTM pixel size (average value ~1.4 m).

- 1. ***Sediment connectivity (IC)***

The topography-based index of sediment connectivity (IC [^24^](https://paperpile.com/c/kZI1KG/QGFr)) represents the potential connection between different parts of a catchment. The considered index is as a refinement of the work by [^25^](https://paperpile.com/c/kZI1KG/jyM1). In particular, IC evaluates the potential connection between hillslopes and the investigated channel, which act as targets for the potentially transported sediment, and it is defined as :

$IC={log}_{10}(\frac{D_{up}}{D_{dn}})$ Eq. 1[^24,26,27^](https://paperpile.com/c/kZI1KG/er4U+QGFr+VuLE)

where D_up_ and D_dn_ are the upslope and downslope components of connectivity respectively. D_up_ represent the potential for downward routing of the sediment available upstream and it depends on the upslope catchment area, mean slope and terrain roughness. D_dn_ takes into account the flow path length that a particle has to travel to arrive at the nearest target or sink and it depends on path length, terrain roughness and gradient along the downslope path. For this work, we applied a surface roughness weighting factor as proposed in [^28^](https://paperpile.com/c/kZI1KG/7ZWk), and for the roughness evaluation, we selected a window (kernel) scale equal to the optimum scale of analysis defined by the fitting-enforcing approach (Dataset S1, Chapter 1.1).

The IC is defined in the range of [−∞, +∞], with connectivity increasing within a watershed for larger IC values. To make the connectivity values comparable across basins, we normalised the IC in a range of 0-1.

The IC maps were further classified into four classes (High, Medium-High, Medium-Low and Low) by identifying breakpoints that best grouped similar values and maximised the differences between classes (natural breaks), as described in [^24,29^](https://paperpile.com/c/kZI1KG/QGFr+2Nkb).

Fig. S2 shows the connectivity maps for the considered catchments, while Dataset S2 includes the classification and some statistics on IC.


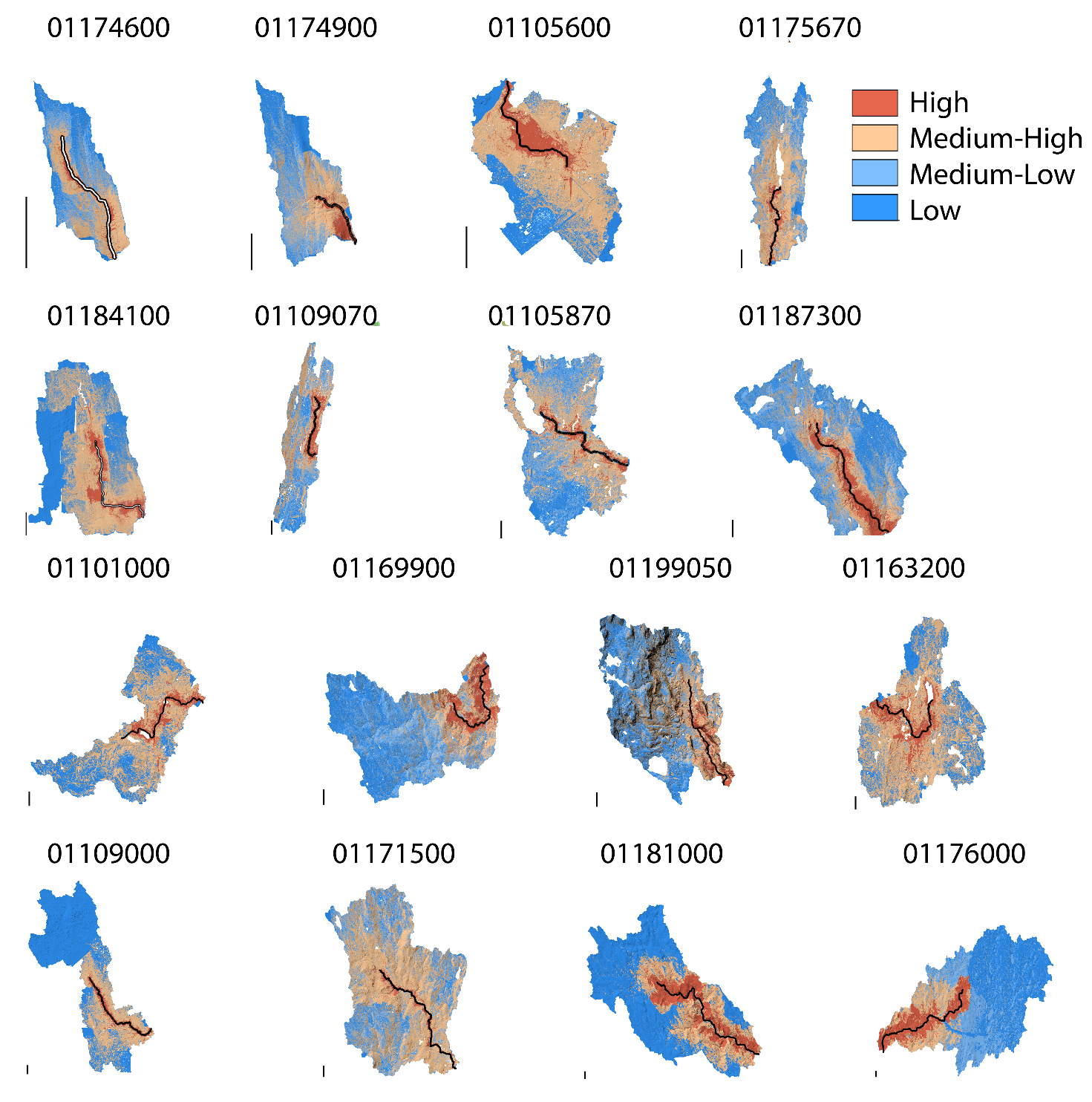


Figure S2: connectivity for the different watersheds, and areas were connectivity is High, Medium-High, Medium-Low and Low. The figure also shows the USGS Station ID and a scalebar at 1km near each watershed. The figure has been arranged using customized scripts in Matlab 2018b [https://www.mathworks.com/release2018b]. Connectivity was evaluated using the software provided by ^27^

- 1. ***Atmosphere: rainfall***

To characterise the rainfall regime, we considered the dataset described in [^30^](https://paperpile.com/c/kZI1KG/Xr4P), based on the concentration index –CI- proposed by [^31^](https://paperpile.com/c/kZI1KG/LY3v)(Fig. S3). The CI is an index which provides information on the frequency distribution of daily precipitation quantities: higher values of CI represent higher cumulated rain within fewer days. A threshold of 0.61 generally distinguishes the values that could be considered high from moderate and low values [^31^](https://paperpile.com/c/kZI1KG/LY3v).

The original dataset has a resolution of 0.25° (~500km^2^ at the latitude and longitude range of the considered watersheds). For each basin, therefore, we calculated an area-weighted average of the CI values (the area weight was represented by the percentage of the catchment area covered by each 0.25° pixel) (Dataset S1).


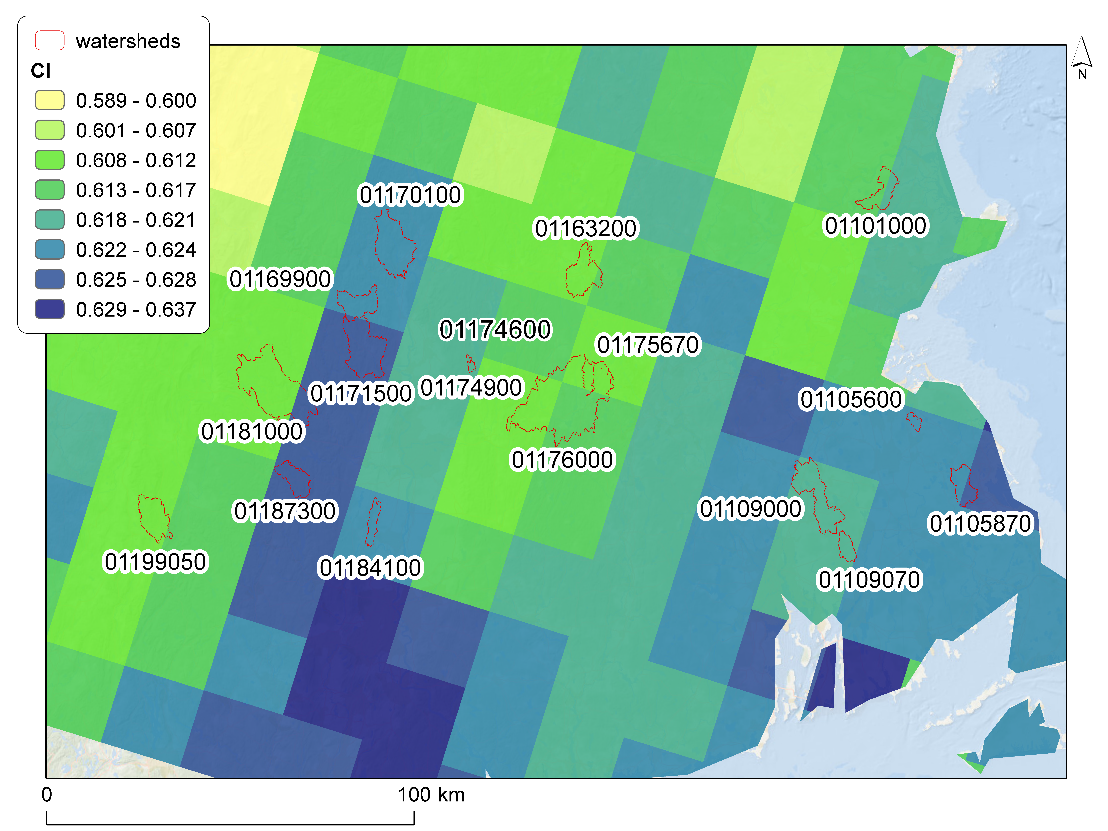


Fig S3: Concentration index (CI) from [^30^](https://paperpile.com/c/kZI1KG/Xr4P). The figure also shows the USGS Station ID for each watershed. The figure has been arranged using ArcGis 10.7 [www.arcgis.com].

- 1. ***Trends on flows and flood hazard***

We estimated the trends in flood hazard as the number of mean daily streamflow values in each year that equalled or exceeded a fixed discharge (set as the 30th and 95th percentile of the flows) for at least 5 consecutive days. In this study, we will refer to the events exceeding the 95^th^ percentile as “floods”, acknowledging that the events identified in this way do not necessarily lead to water outside the river banks.

As suggested by [^32^](https://paperpile.com/c/kZI1KG/5CQn) we only calculated flow frequency for calendar years with nearly complete data (≥ 350 mean daily streamflow values).

To characterise long-term trends in flow frequency, we followed the procedure of [^32^](https://paperpile.com/c/kZI1KG/5CQn). Using nonlinear least squares, we fitted an exponential trend to the flow frequency time series (on linear axes, in order to preserve values of F=0), as shown in Eq. 2 and Fig. S4.

$\frac{FF}{mean(FF)}=\frac{exp(r\Delta t)}{mean(\exp\left( r\Delta t \right))}$ Eq. 2

where mean(F) is the mean of the observed flow exceedance, r is the fractional change in flow frequency per unit of time, and Δt = date–mean(date). If Δt is expressed in decades, then 100 times the coefficient r yields the rate of change in flow exceedance in percent per decade, as reported in Fig. S4.

Following the procedure by [^32^](https://paperpile.com/c/kZI1KG/5CQn), we used a Monte Carlo permutation method to assess the significance of these trends, because permutation tests require no assumptions on the underlying distribution.


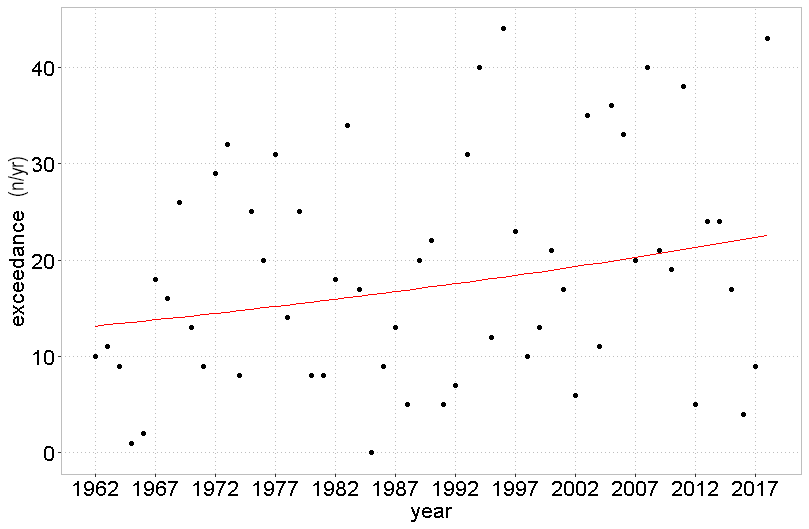


Figure S4. Flow frequency trend (numbers of events exceeding the selected threshold for more than 5 consecutive days, n/yr) versus year at Salmon Creek at Lime Rock, CT (USGS id 01199050). Dark circles represent computed values of Q95 exceedance (number of events with mean daily discharge equaling or exceeding the long-term Q95 for more than 5 consecutive days each year). The red exponential curve represents the mean-unbiased exponential trend in annual exceedance. This site has a mean number of events of 19 per year, and a trend in frequency of 8% per decade.

***Dataset S1:*** Scale, bankfull properties, network properties and climate for the considered watersheds. USGS ID, name, declared drainage area, measured bankfull width, bankfull discharge and return period of bankfull discharge at the outlet^23^, Drainage density, the drainage frequency expressed in number of streams per unit of area (#/km^2^), watershed order, length of the investigated stream, the distance from the farthest channel head to the outlet along the network, watershed-scale and area-weighted CI for each watershed. Readers should refer to table 1 in the main manuscript for a description of the acronyms.

| ID | Name | | A (km^2^) | W_o_ (m) | QQ_bkf_ (m^3^/s) | RPQQ_bkf_ (yr) | D_d_  (km/km^2^) | D_f_ (#/km^2^) | Ω | L_a_  (km) | L_l_  (km) | Watershed Scale (m) | CI |
| --- | --- | --- | --- | --- | --- | --- | --- | --- | --- | --- | --- | --- | --- |
| 01174600 | CADWELL CREEK NEAR PELHAM, MA | 1.49 | | **4.11** | 0.68 | 1.24 | 2.10 | 1.22 | 2 | 2.12 | 2.12 | 18.23 | 0.618 |
| 01174900 | CADWELL CREEK NEAR BELCHERTOWN, MA | 6.33 | | 5.67 | 1.30 | 1.03 | 1.64 | 0.45 | 3 | 2.16 | 4.69 | 16.04 | 0.617 |
| 01184100 | STONY BROOK NEAR WEST SUFFIELD, CT. | 26.73 | | 13.35 | 7.76 | 1.38 | 0.95 | 0.11 | 3 | 5.56 | 7.88 | 28.91 | 0.624 |
| 01187300 | HUBBARD RIVER NR. WEST HARTLAND, CT. | 50.59 | | 22.33 | 14.36 | 1.21 | 0.91 | 0.06 | 3 | 9.53 | 12.73 | 17.06 | 0.626 |
| 01199050 | SALMON CREEK AT LIME ROCK, CT. | 72.96 | | 14.09 | 13.98 | 1.52 | 0.57 | 0.04 | 3 | 10.83 | 13.22 | 5.91 | 0.609 |
| 01175670 | SEVENMILE RIVER NEAR SPENCER, MA | 22.24 | | 9.66 | 4.62 | 1.74 | 1.22 | 0.13 | 3 | 5.50 | 9.94 | 15.49 | 0.611 |
| 01101000 | PARKER RIVER AT BYFIELD, MA | 52.88 | | 14.16 | 7.73 | 3.16 | 1.01 | 0.07 | 4 | 9.96 | 13.30 | 6.69 | 0.617 |
| 01109070 | SEGREGANSET RIVER NEAR DIGHTON, MA | 24.79 | | 9.92 | 5.46 | 1.07 | 0.69 | 0.15 | 4 | 5.40 | 7.96 | 31.69 | 0.620 |
| 01105870 | JONES RIVER AT KINGSTON, MA | 45.30 | | 11.78 | 7.16 | 3.48 | 0.96 | 0.08 | 4 | 7.57 | 9.60 | 30.24 | 0.624 |
| 01163200 | OTTER RIVER AT OTTER RIVER, MA | 82.78 | | 18.19 | 10.59 | 1.43 | 0.60 | 0.03 | 3 | 10.83 | 12.81 | 17.44 | 0.608 |
| 01169900 | SOUTH RIVER NEAR CONWAY, MA | 60.99 | | 19.97 | 48.42 | 1.71 | 0.46 | 0.06 | 4 | 8.81 | 10.69 | 14.84 | 0.623 |
| 01105600 | OLD SWAMP RIVER NEAR SOUTH WEYMOUTH, MA | 8.79 | | 10.84 | 6.40 | 2.73 | 1.01 | 0.20 | 2 | 3.44 | 4.51 | 8.16 | 0.622 |
| 01171500 | MILL RIVER AT NORTHAMPTON, MA | 126.97 | | 25.76 | 45.31 | 1.33 | 0.52 | 0.03 | 4 | 15.18 | 17.63 | 15.85 | 0.624 |
| 01109000 | WADING RIVER NEAR NORTON, MA | 93.51 | | 12.39 | 8.35 | 1.15 | 0.38 | 0.04 | 4 | 13.09 | 15.74 | 33.40 | 0.620 |
| 01176000 | QUABOAG RIVER AT WEST BRIMFIELD, MA | 376.52 | | 40.37 | 28.60 | 1.54 | 0.21 | 0.01 | 4 | 25.96 | 28.05 | 12.39 | 0.613 |
| 01181000 | WEST BRANCH WESTFIELD RIVER AT HUNTINGTON, MA | 238.99 | | 37.84 | 98.25 | 1.34 | 0.62 | 0.02 | 4 | 23.58 | 26.53 | 11.92 | 0.612 |

***Dataset S2:*** Flows, Connectivity, and HSF parameters of the considered watersheds. USGS ID, name, coefficient of variations of flows, mean discharge, low and high flows, coefficient of variation of connectivity, mean connectivity, low and high connectivity, and coefficients of HSF. Readers should refer to table 1 in the main manuscript for a description of the acronyms.

| ID | Q_cv_ | Q_mean_ (m^3^/s/km^2^) | Q_30_  (m^3^/s/km^2^) | Q_95_  (m^3^/s/km^2^) | IC_cv_ | IC_mean_ | IC_30_ | IC_95_ | IC_L%_ | IC_H%_ | α | β |
| --- | --- | --- | --- | --- | --- | --- | --- | --- | --- | --- | --- | --- |
| 01174600 | 0.59 | 0.03 | 0.01 | 0.12 | 0.29 | 0.27 | 0.49 | 0.29 | 54.55 | 45.45 | 3.44 | 0.29 |
| 01174900 | 0.63 | 0.14 | 0.04 | 0.48 | 0.25 | 0.29 | 0.49 | 0.25 | 58.86 | 41.14 | 2.48 | 0.26 |
| 01184100 | 0.56 | 0.56 | 0.12 | 2.03 | 0.15 | 0.39 | 0.54 | 0.15 | 30.85 | 69.15 | 7.63 | 0.15 |
| 01187300 | 0.51 | 1.18 | 0.24 | 4.25 | 0.25 | 0.26 | 0.46 | 0.25 | 43.91 | 56.09 | 4.09 | 0.40 |
| 01199050 | 0.82 | 1.46 | 0.57 | 4.28 | 0.14 | 0.36 | 0.50 | 0.14 | 44.32 | 55.68 | 4.55 | 0.23 |
| 01175670 | 0.74 | 0.43 | 0.11 | 1.39 | 0.20 | 0.29 | 0.45 | 0.20 | 49.27 | 50.73 | 3.05 | 0.30 |
| 01101000 | 0.78 | 1.07 | 0.25 | 3.48 | 0.16 | 0.39 | 0.56 | 0.16 | 52.92 | 47.08 | 4.70 | 0.22 |
| 01109070 | 0.60 | 0.63 | 0.12 | 2.17 | 0.16 | 0.39 | 0.56 | 0.16 | 61.04 | 38.96 | 3.01 | 0.47 |
| 01105870 | 1.13 | 0.96 | 0.45 | 2.61 | 0.16 | 0.36 | 0.51 | 0.16 | 38.24 | 61.76 | 4.79 | 0.28 |
| 01163200 | 0.92 | 1.81 | 0.68 | 5.49 | 0.15 | 0.34 | 0.48 | 0.15 | 67.61 | 32.39 | 1.75 | 0.56 |
| 01169900 | 0.62 | 1.61 | 0.47 | 5.13 | 0.19 | 0.30 | 0.46 | 0.19 | 52.20 | 47.80 | 9.45 | 0.15 |
| 01105600 | 0.57 | 0.25 | 0.07 | 0.82 | 0.18 | 0.40 | 0.59 | 0.18 | 26.63 | 73.37 | 0.92 | 0.96 |
| 01171500 | 0.70 | 2.88 | 0.85 | 9.29 | 0.14 | 0.33 | 0.45 | 0.14 | 63.70 | 36.30 | 2.24 | 0.48 |
| 01109000 | 0.90 | 2.09 | 0.59 | 6.37 | 0.14 | 0.40 | 0.56 | 0.14 | 34.24 | 65.76 | 2.51 | 0.37 |
| 01176000 | 0.95 | 7.16 | 2.58 | 20.81 | 0.12 | 0.47 | 0.62 | 0.12 | 56.45 | 43.55 | 1.70 | 0.53 |
| 01181000 | 0.58 | 5.61 | 1.39 | 19.37 | 0.16 | 0.39 | 0.56 | 0.16 | 70.59 | 29.41 | 1.73 | 0.55 |

**References**

1. [Sofia, G.](http://paperpile.com/b/kZI1KG/T5za) , Tarolli, P., Cazorzi, F. & Dalla Fontana, G. [An objective approach for feature extraction: distribution analysis and statistical descriptors for scale choice and channel network identification. *Hydrol. Earth Syst. Sci.* **15**, 1387/1402 (2011).](http://paperpile.com/b/kZI1KG/T5za)

2. [Mutzner, R., Tarolli, P., Sofia, G., Parlange, M. B. & Rinaldo, A. Field study on drainage densities and rescaled width functions in a high-altitude alpine catchment. *Hydrol. Process.* **30**, 2138–2152 (2016).](http://paperpile.com/b/kZI1KG/iRYE)

3. [Evans, I. S., Young, M. & Gill, J. S. An integrated system of terrain analysis and slope mapping, final report. *Univ. of Durham, Durham, NC* (1979).](http://paperpile.com/b/kZI1KG/MLm9)

4. [Yokoyama, R., Shirasawa, M. & Pike, R. J. Visualizing topography by openness: A new application of image processing to digital elevation models. *Photogrammetric Engineering & Remote Sensing* **68**, 257–265 (2002).](http://paperpile.com/b/kZI1KG/KNS4)

5. [LoRe, G., Fuller, I.C., Sofia, G., Tarolli, P. High-resolution mapping of Manawatu palaeochannels. *N. Z. Geog.* **74**(2), 77-91, (2018) doi:](http://paperpile.com/b/kZI1KG/IGBm)[10.1111/nzg.12186](http://dx.doi.org/10.1111/nzg.12186)[.](http://paperpile.com/b/kZI1KG/IGBm)

6. [Sofia, G., Tarolli, P., Cazorzi, F. & Dalla Fontana, G. Downstream hydraulic geometry relationships: Gathering reference reach-scale width values from LiDAR. *Geomorphology*  **250**, 236–248 (2015).](http://paperpile.com/b/kZI1KG/8pC4)

7. [Sofia, G., Di Stefano, C., Ferro, V. & Tarolli, P. Morphological Similarity of Channels: From Linear Erosional Features (Rill, Gully) to Alpine Rivers. *Land Degrad. Dev.* **28**, 1717–1728 (2017).](http://paperpile.com/b/kZI1KG/FKj8)

8. [Quinn, P., Beven, K., Chevallier, P. & Planchon, O. The prediction of hillslope flow paths for distributed hydrological modelling using digital terrain models. *Hydrol. Process.* **5**, 59–79 (1991).](http://paperpile.com/b/kZI1KG/RXwB)

9. [Singh, V. P., Yang, C. T. & Deng, Z. Q. Downstream hydraulic geometry relations: 1. Theoretical development. *Water Resour. Res.* **39**, (2003).](http://paperpile.com/b/kZI1KG/pSQIs)

10. [Singh, V. P., Yang, C. T. & Deng, Z.-Q. Downstream hydraulic geometry relations: 2. Calibration and testing. *Water Resources Research* vol. 39 (2003).](http://paperpile.com/b/kZI1KG/HlaY6)

11. [Julien, P. Y. Downstream hydraulic geometry of alluvial rivers. in *IAHS-AISH Proceedings and Reports* vol. 367 3–11 (2014).](http://paperpile.com/b/kZI1KG/EEQDx)

12. [Leopold L.B. Maddock, T., Jr. *The hydraulic geometry of stream channels and some physiographic implications*. 57 (1953).](http://paperpile.com/b/kZI1KG/gRztm)

13. [Wolman, M. G. & Leopold, L. B. *River flood plains: some observations on their formation*. vols 282-C (1957).](http://paperpile.com/b/kZI1KG/yKyNc)

14. [Leopold L.B. *A View of the River*. (Harvard University Press, Cambridge, Massachussetts, 1994).](http://paperpile.com/b/kZI1KG/YzTOx)

15. [Leopold L.B., Wolman, M. G. & Miller, J. P. *Fluvial processes in geomorphology, 522 pp*. 522 (San Francisco, Freeman, 1964).](http://paperpile.com/b/kZI1KG/D9ePp)

16. [Gleason, C. J. Hydraulic geometry of natural rivers: A review and future directions. *Prog. Phys. Geogr.* **39**, 337–360 (2015).](http://paperpile.com/b/kZI1KG/uBtDO)

17. [Wohl, E. Limits of downstream hydraulic geometry. *Geology* **32** , 897–900 (2004).](http://paperpile.com/b/kZI1KG/eo2Tp)

18. [Bieger, K., Rathjens, H., Allen, P. M. & Arnold, J. G. Development and Evaluation of Bankfull Hydraulic Geometry Relationships for the Physiographic Regions of the United States. **51**, 842–858 (2015).](http://paperpile.com/b/kZI1KG/tqJH)

19. [Galster, J. C. Natural and anthropogenic influences on the scaling of discharge with drainage area for multiple watersheds. *Geosphere* **3**, 260 (2007).](http://paperpile.com/b/kZI1KG/tpJd)

20. [Passalacqua, P., Do Trung, T., Foufoula-Georgiou, E., Sapiro, G. & Dietrich, W. E. A geometric framework for channel network extraction from lidar: Nonlinear diffusion and geodesic paths. *Journal of Geophysical Research: Earth Surface* **115**, F01002 (2010).](http://paperpile.com/b/kZI1KG/JpSB)

21. [Lashermes, B., Foufoula-Georgiou, E. & Dietrich, W. E. Channel network extraction from high resolution topography using wavelets. *Geophys. Res. Lett.* **34**, L23S04 (2007).](http://paperpile.com/b/kZI1KG/Fub9)

22. [Cavalli, M. & Tarolli, P. Application of LiDAR technology for rivers analysis. *Italian Journal of Engineering Geology and Environment* 33–34 (2011).](http://paperpile.com/b/kZI1KG/pZ5x)

23. [Bent, G. C. & Waite, A. M. *Equations for Estimating Bankfull Channel Geometry and Discharge for Streams in Massachusetts*. 62pp (2013).](http://paperpile.com/b/kZI1KG/EEkW)

24. [Cavalli, M., Trevisani, S., Comiti, F. & Marchi, L. Geomorphometric assessment of spatial sediment connectivity in small Alpine catchments. *Geomorphology*  **188**, 31–41 (2013).](http://paperpile.com/b/kZI1KG/QGFr)

25. [Borselli, L., Cassi, P. & Torri, D. Prolegomena to sediment and flow connectivity in the landscape: A GIS and field numerical assessment. *Catena* **75**, 268–277 (2008).](http://paperpile.com/b/kZI1KG/jyM1)

26. [Crema, S., Schenato, L., Goldin, B., Marchi, L. & Cavalli, M. Toward the development of a stand-alone application for the assessment of sediment connectivity. *Rendiconti online della Società Geologica Italiana* **34**, 58–61 (2015).](http://paperpile.com/b/kZI1KG/er4U)

27. [Crema, S. & Cavalli, M. SedInConnect: a stand-alone, free and open source tool for the assessment of sediment connectivity. *Comput. Geosci.* **111**, 39–45 (2018).](http://paperpile.com/b/kZI1KG/VuLE)

28. [Cavalli, M., Tarolli, P., Marchi, L. & Dalla Fontana, G. The effectiveness of airborne LiDAR data in the recognition of channel-bed morphology. *Catena* **73**, 249–260 (2008).](http://paperpile.com/b/kZI1KG/7ZWk)

29. [Tarolli, P. & Sofia, G. Human topographic signatures and derived geomorphic processes across landscapes. *Geomorphology*  **255**, 140–161 (2016).](http://paperpile.com/b/kZI1KG/2Nkb)

30. [Royé, D. & Martin-Vide, J. Concentration of daily precipitation in the contiguous United States. *Atmos. Res.* **196**, 237–247 (2017).](http://paperpile.com/b/kZI1KG/Xr4P)

31. [Martin-Vide, J. Spatial distribution of a daily precipitation concentration index in peninsular Spain. *Int. J. Climatol.* **24**, 959–971 (2004).](http://paperpile.com/b/kZI1KG/LY3v)

32. [Slater, L. J., Singer, M. B. & Kirchner, J. W. Hydrologic versus geomorphic drivers of trends in flood hazard. *Geophys. Res. Lett.* **42**, 370–376 (2015).](http://paperpile.com/b/kZI1KG/5CQn)
